# Lithium and sodium 3-(3,4-di­hydroxy­phen­yl)propenoate hydrate

**DOI:** 10.1107/S2056989024002494

**Published:** 2024-03-26

**Authors:** Irén Bieler, Christoph Wagner, Kurt Merzweiler

**Affiliations:** a Martin-Luther-Universität Halle Wittenberg, Naturwissenschaftliche Fakultät II, Institut für Chemie, Germany; Vienna University of Technology, Austria

**Keywords:** crystal structure, caffeic acid, lithium, sodium, hydrogen-bonding

## Abstract

Treatment of 3-(3,4-di­hydroxy­phen­yl)propenoic acid (caffeic acid or 3,4-di­hydroxy­cinnamic acid) with the alkali hydroxides *M*OH (*M* = Li, Na) in aqueous solution led to the formation of poly[aqua­[μ-3-(3,4-di­hydroxy­phen­yl)propenoato]lithium], [Li(C_9_H_7_O_4_)(H_2_O)]_*n*_, **1**, and poly[aqua­[μ-3-(3,4-di­hydroxy­phen­yl)propenoato]sodium], [Na(C_9_H_7_O_4_)(H_2_O)]_*n*_, **2**. The crystal structure of **1** consists of a lithium cation that is coordinated nearly tetra­hedrally by three carboxyl­ate oxygen atoms and a water mol­ecule. The carboxyl­ate groups adopt a μ_3_-κ^3^ *O*:*O*′:*O*′ coordination mode that leads to a chain-like catenation of Li cations and carboxyl­ate units parallel to the *b* axis. Moreover, the lithium carboxyl­ate chains are connected by hydrogen bonds between water mol­ecules attached to lithium and catechol OH groups. The crystal structure of **2** shows a sevenfold coordination of the sodium cation by one water mol­ecule, two monodentately binding carboxyl­ate groups and four oxygen atoms from two catechol groups. The coordination polyhedra are linked by face- and edge-sharing into chains extending parallel to the *b* axis. The chains are inter­linked by the bridging 3-(3,4-di­hydroxy­phen­yl)propenoate units and by inter­molecular hydrogen bonds to form the tri-periodic network.

## Abstract 2

The Li cation in the crystal structure of the lithium salt LiC_9_H_7_O_4_·H_2_O shows a coordination number of four whereas the Na cation in the crystal structure of the sodium salt NaC_9_H_7_O_4_·H_2_O shows a coordination number of seven.

## 1. Chemical context

*trans-*3-(3,4-Di­hydroxy­phen­yl)-2-propenoic acid (caffeic acid) is ubiquitous in plants and plays a role as an inter­mediate in the biosynthesis of lignin (Boerjan *et al.*, 2003 ▸). The first X-ray crystal-structure analysis of caffeic acid dates back to the year 1987 (García-Granda *et al.*, 1987 ▸), and a more recent study was published in 2015 (Kumar *et al.*, 2015 ▸). In current research, caffeic acid is used as a co-crystallizing agent, particularly for pharmaceutically relevant compounds such as 5-fluoro­uracil (Yu *et al.*, 2020 ▸). The simultaneous presence of the carboxyl and the catechol moieties renders caffeic acid a versatile ligand in coordination chemistry, in particular after deprot­on­ation of the acidic groups (Petrou *et al.*, 1993 ▸). However, transition-metal complexes of caffeic acid derivatives have not yet been structurally investigated. Even for simple alkali metal caffeates, reports are rare and, up to now, only potassium caffeate has been studied in detail as the potassium caffeate/caffeic acid co-crystallization product (Lombardo *et al.*, 2011 ▸). 
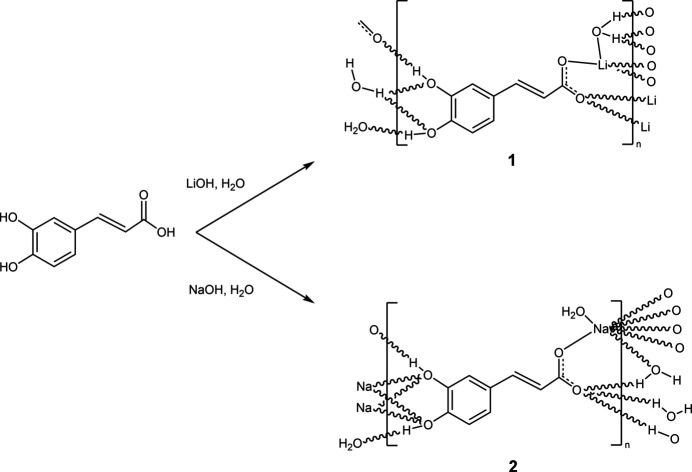


Here we report the crystallization and crystal-structure analysis of the lithium and sodium salts of caffeic acid, LiC_9_H_7_O_4_·H_2_O, **1**, and NaC_9_H_7_O_4_·H_2_O, **2**, respectively.

## 2. Structural commentary

**Figure 1:**
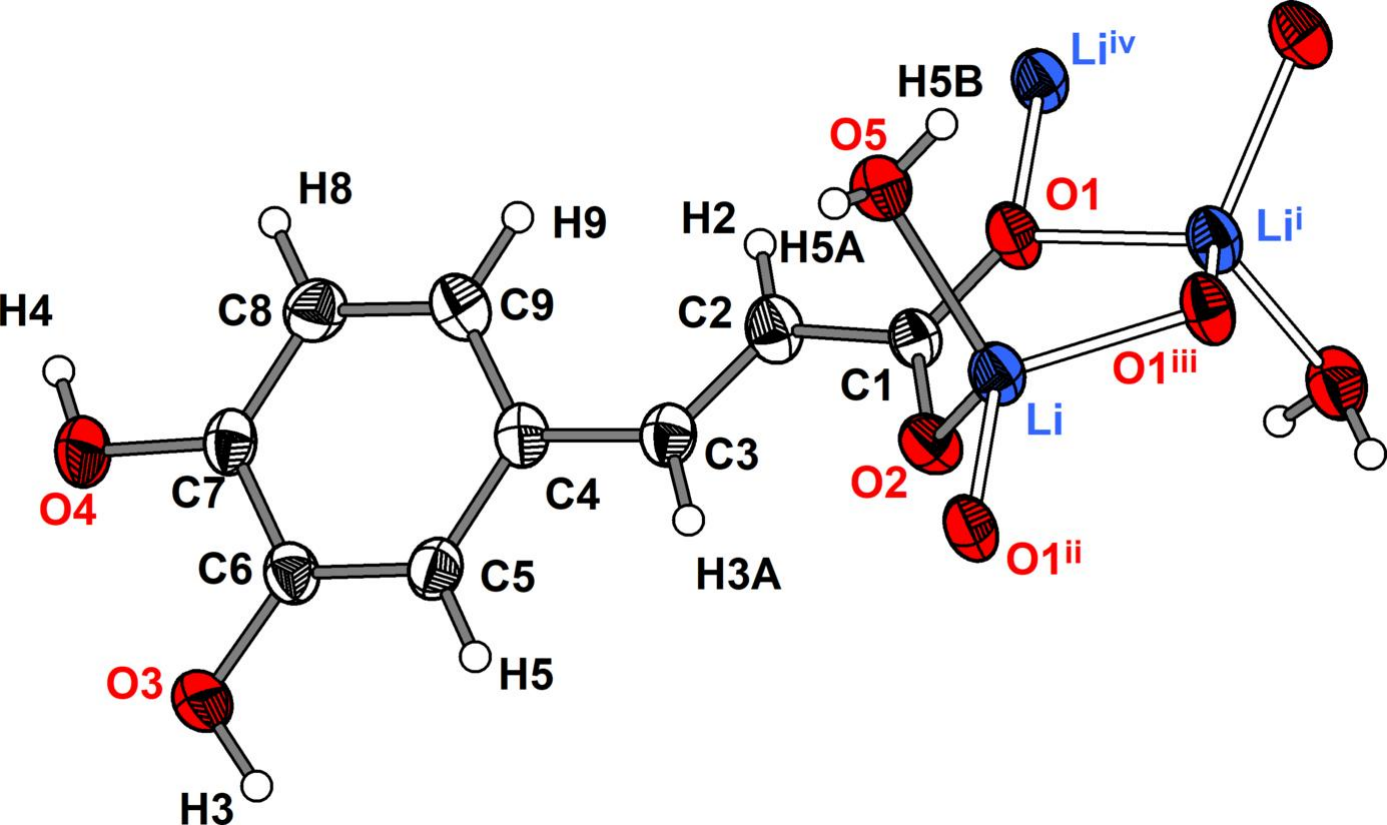
Mol­ecular structure of the (3,4-di­hydroxy­phen­yl)propenoate anion in compound **1** along with the coordination sphere of the lithium cation. The asymmetric unit is displayed with filled bonds. Displacement ellipsoids are drawn at the 50% probability level and H atoms are shown as small spheres of arbitrary radii. Symmetry codes refer to Table 1 ▸.

**Figure 2:**
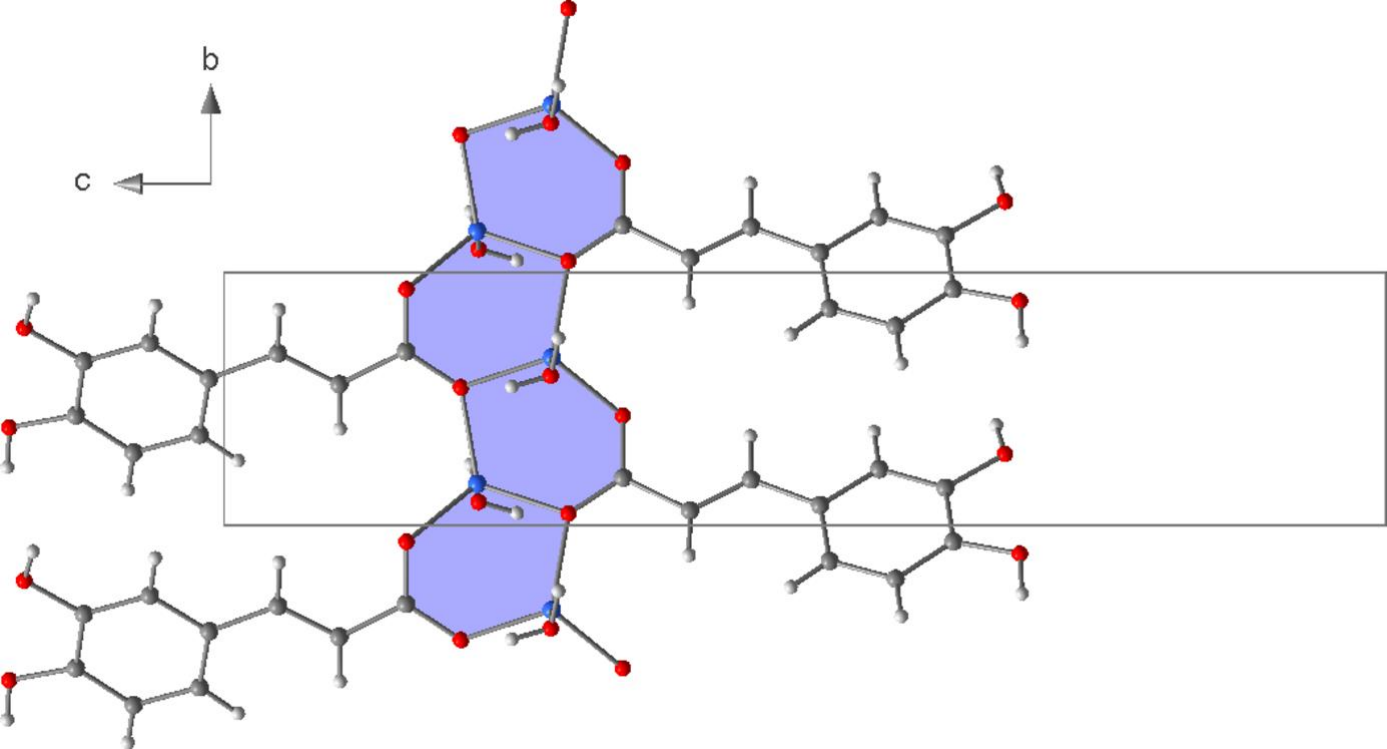
Section of the crystal structure of **1** showing the six-membered Li_2_O_3_C rings.

**Figure 3:**
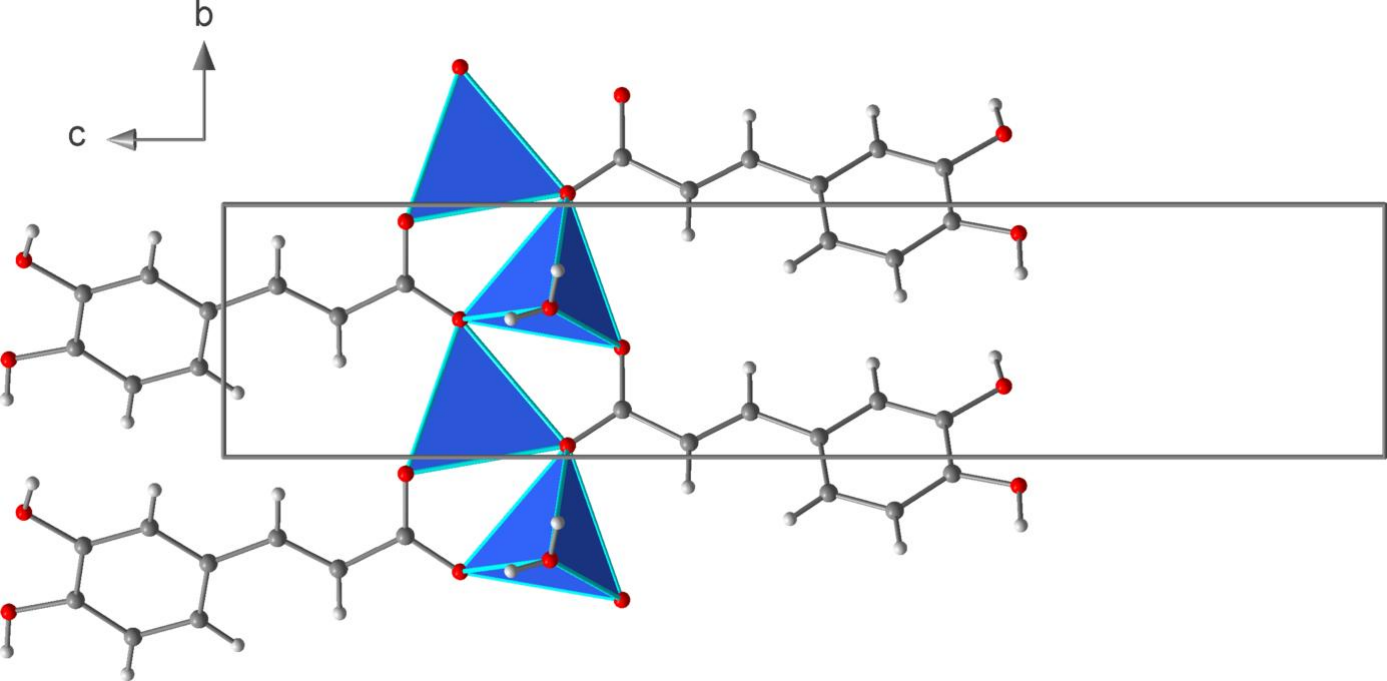
Section of the crystal structure of **1** showing the linkage of the LiO_4_ tetra­hedra.

The asymmetric unit of **1** comprises one Li cation, one 3-(3,4-di­hydroxy­phen­yl)propenoate anion and one water mol­ecule (Fig. 1 ▸). The Li cation is coordinated nearly tetra­hedrally by three carboxyl­ate O atoms of three caffeate anions and one water mol­ecule. The Li—O distances range from 1.908 (2) to 2.005 (3) Å and the O—Li—O angles from 105.35 (12) to 112.20 (11)° (Table 1 ▸). These values are similar to those reported for other lithium carboxyl­ate compounds such as lithium acetate monohydrate [Li—O: 1.920 (2) to 2.031 (2) Å, O—Li—O: 99.78 (10) to 124.21 (11)°; Martínez Casado *et al.*, 2011 ▸]. The carboxyl­ate group adopts a *μ*_3_-*κ* ^3^O:O′,O′ coordination mode. This leads to the formation of six-membered Li_2_O_3_C rings that are catenated parallel to the *b* axis by edge-sharing (Fig. 2 ▸). Alternatively, the chain structure can be derived from condensation of corner-sharing LiO_4_ tetra­hedra (Fig. 3 ▸). The translational period of the 2_1_-type helix corresponds to the length of the *b* axis and one repeating unit comprises two LiO_4_ tetra­hedra. Chains of corner-sharing LiO_4_ tetra­hedra are not unusual for lithium carboxyl­ate monohydrates, and similar patterns were observed for a lithium chloride glycine adduct (Müller *et al.*, 1994 ▸) and lithium 2,4,6-tri­fluoro­benzoate hydrate (Lamann *et al.*, 2012 ▸), which may serve as representative examples.

**Figure 4:**
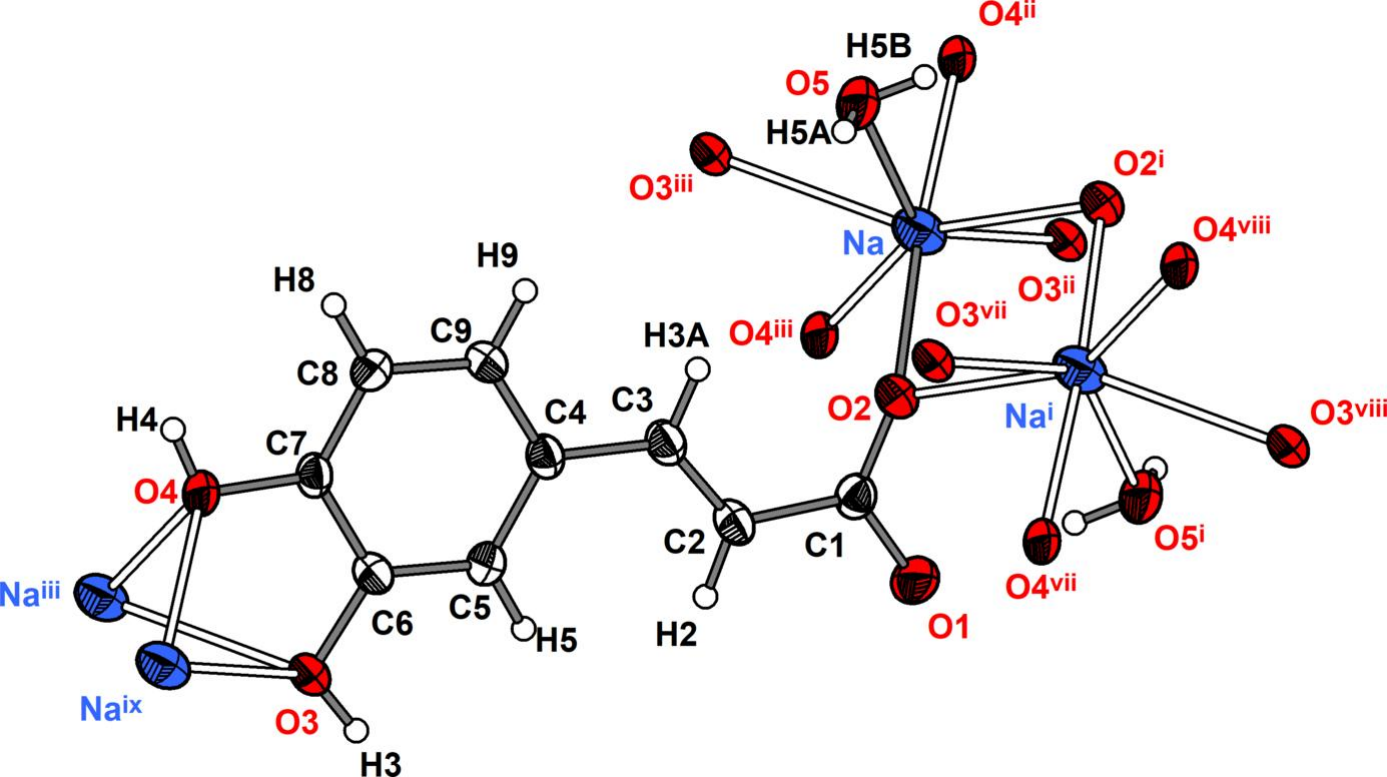
Mol­ecular structure of the caffeate anion in compound **2** along with the coordination sphere of the sodium cation. The asymmetric unit is displayed with filled bonds. Displacement ellipsoids are drawn at the 50% probability level and H atoms are shown as small spheres of arbitrary radii. Symmetry codes refer to Table 2 ▸. Additional symmetry codes: (vii) −*x* + 1, −*y*, −*z* + 1; (viii) *x*, *y* − 1, *z* + 1; (ix) *x*, *y*, *z* − 1.

**Figure 5:**
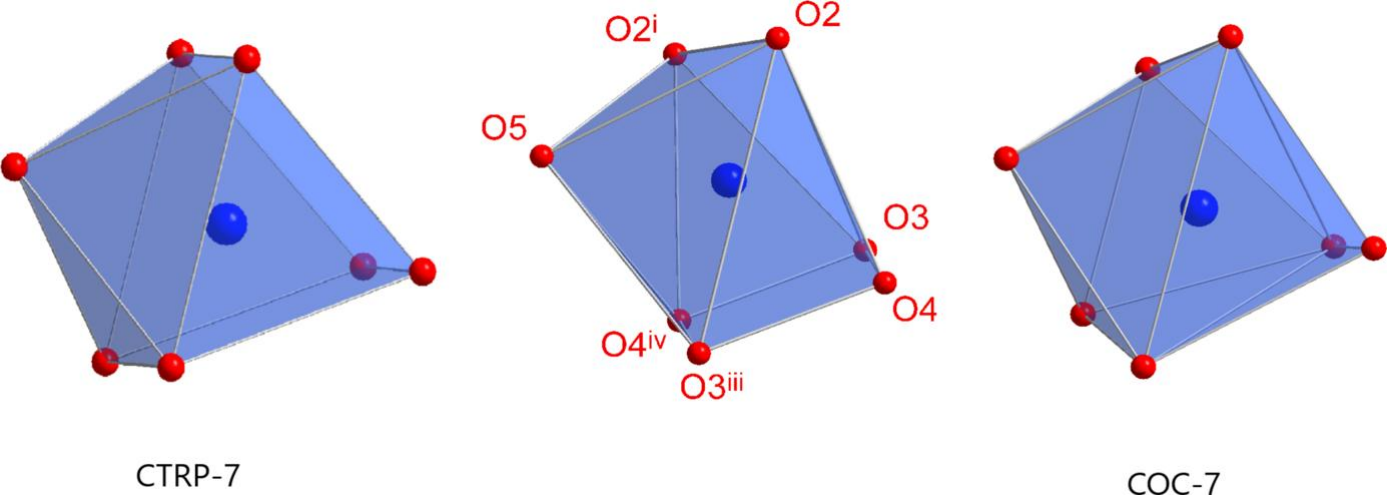
Representation of the NaO_7_ polyhedron in **2** (centre) and the best fitting regular polyhedra TRP-7 (left) and COC-7 (right).

In the crystal structure of **2**, the sodium cation adopts a sevenfold coordination from one water oxygen atom, two carboxyl­ate and four catechol oxygen atoms (Fig. 4 ▸). According to a SHAPE analysis (*SHAPE 2.1*; Llunell *et al.*, 2013 ▸), the NaO_7_ polyhedron is roughly related to the face-capped octa­hedron (CShM 2.807) and to the face-capped trigonal prism (CShM 3.593) with some preference to the former (Llunell *et al.*, 2013 ▸; Pinsky & Avnir, 1998 ▸; Casanova *et al.*, 2004 ▸; Cirera *et al.*, 2005 ▸). Numerical data for this analysis are given in Table 2 ▸. The centre of Fig. 5 ▸ displays the observed NaO_7_ polyhedron and the idealized CTRP-7 (left) and COC-7 (right) polyhedra in order to illustrate the degree of distortion. The Na—O distances are in the range from 2.3185 (14) to 2.7897 (17) Å (Table 3 ▸) and are comparable to those found in monosodium tartrate hydrate [2.3331 (12) to 2.6740 (12) Å], which also displays coordination number seven for the sodium cation (Al-Dajani *et al.*, 2010 ▸). Generally, sodium carboxyl­ates with coordination number seven for the cation are rather rare. A search of the Cambridge Structural Database (CSD, version 2022.5.43; Groom *et al.*, 2016 ▸) gave 20 matches. In this selection, the Na—O distances range from 2.299 to 3.017 Å with a median value of 2.44 Å (lower quartile: 2.380 Å, upper quartile: 2.557 Å). Regarding the coordination mode of the carboxyl­ate unit, the sodium salt **2** differs from the lithium salt **1** in the way that only one carboxyl­ate O atom (O2) is involved in coordination. Furthermore, the coordination mode of the catechol groups is also different in the two structures. In the case of **1**, the catechol groups are part of the hydrogen-bonding network and there is no direct Li—O coordination from these groups. In contrast, the crystal structure of **2** reveals a direct coordination by the catechol oxygen atoms. Here, the catechol group acts as a chelating ligand and connects two sodium cations. The coordination mode can be described as *μ-κ* ^4^ *O*,*O*′:*O*,*O*′. Up to now, sodium compounds with chelating catechol groups have been observed only rarely. The CSD database search resulted in four structures with this coordination motif, *e.g*. in sodium quercetin-5′-sulfonate acetone solvate (Maciołek *et al.*, 2022 ▸).

**Figure 6:**
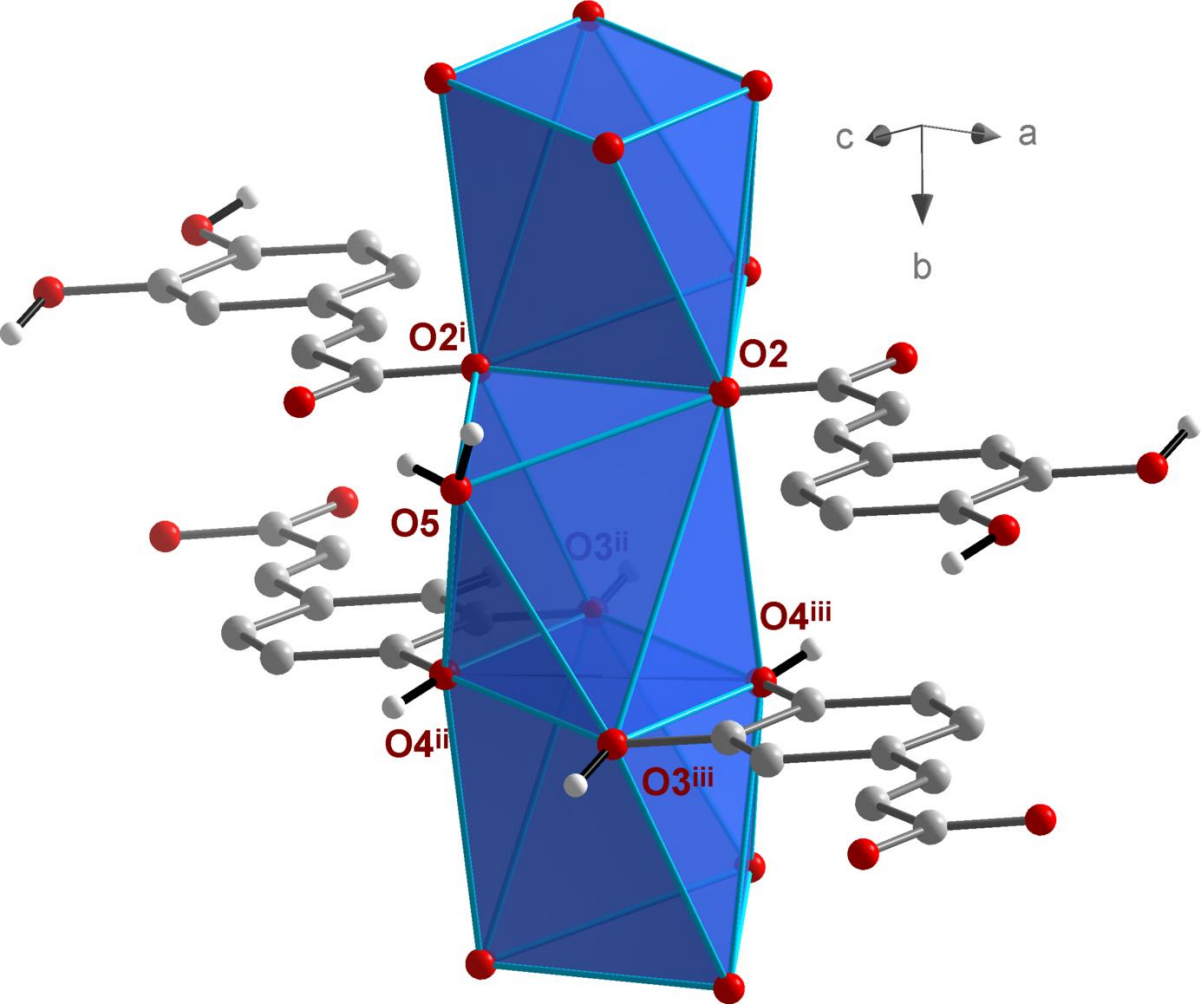
Linkage of the NaO_7_ polyhedra by edge- and face-sharing in the crystal structure of **2**.

**Figure 7:**
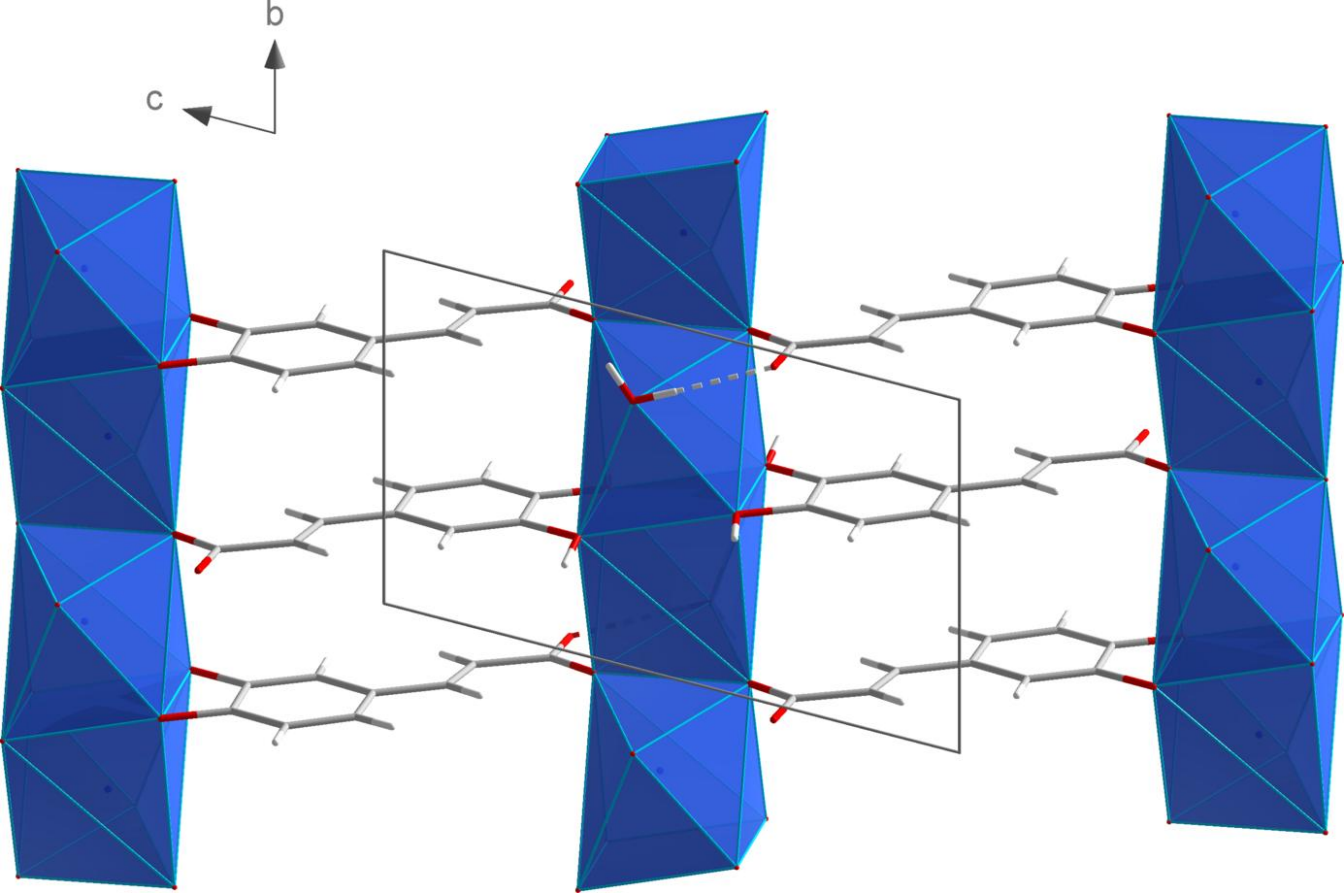
Linkage of the NaO_7_ polyhedral chains by (3,4-di­hydroxy­phen­yl)propenoate units in the crystal structure of **2**.

The NaO_7_ polyhedra are linked by edge- (O2 and O2^i^) and face- (O3^iii^, O4^iii^, O3^iv^, O4^iv^) sharing to give chains propagating parallel to the *b* axis (Fig. 6 ▸). Additional inter­linking of these chains by *μ*_4_-bridging (3,4-di­hydroxy­phen­yl)propenoate units (Fig. 7 ▸) leads to layers extending parallel to the *bc* plane.

In the structures of **1** and **2**, the bond lengths and angles within the 3-(3,4-di­hydroxy­phen­yl)propenoate anions are very similar (Tables 1 ▸ and 3 ▸). The anions are nearly planar apart from a slight tilt [**1**: 6.3 (2)°, **2**: 1.4 (2)°] of the carboxyl­ate group along the C1—C2 bond.

## 3. Supra­molecular features

**Figure 8:**
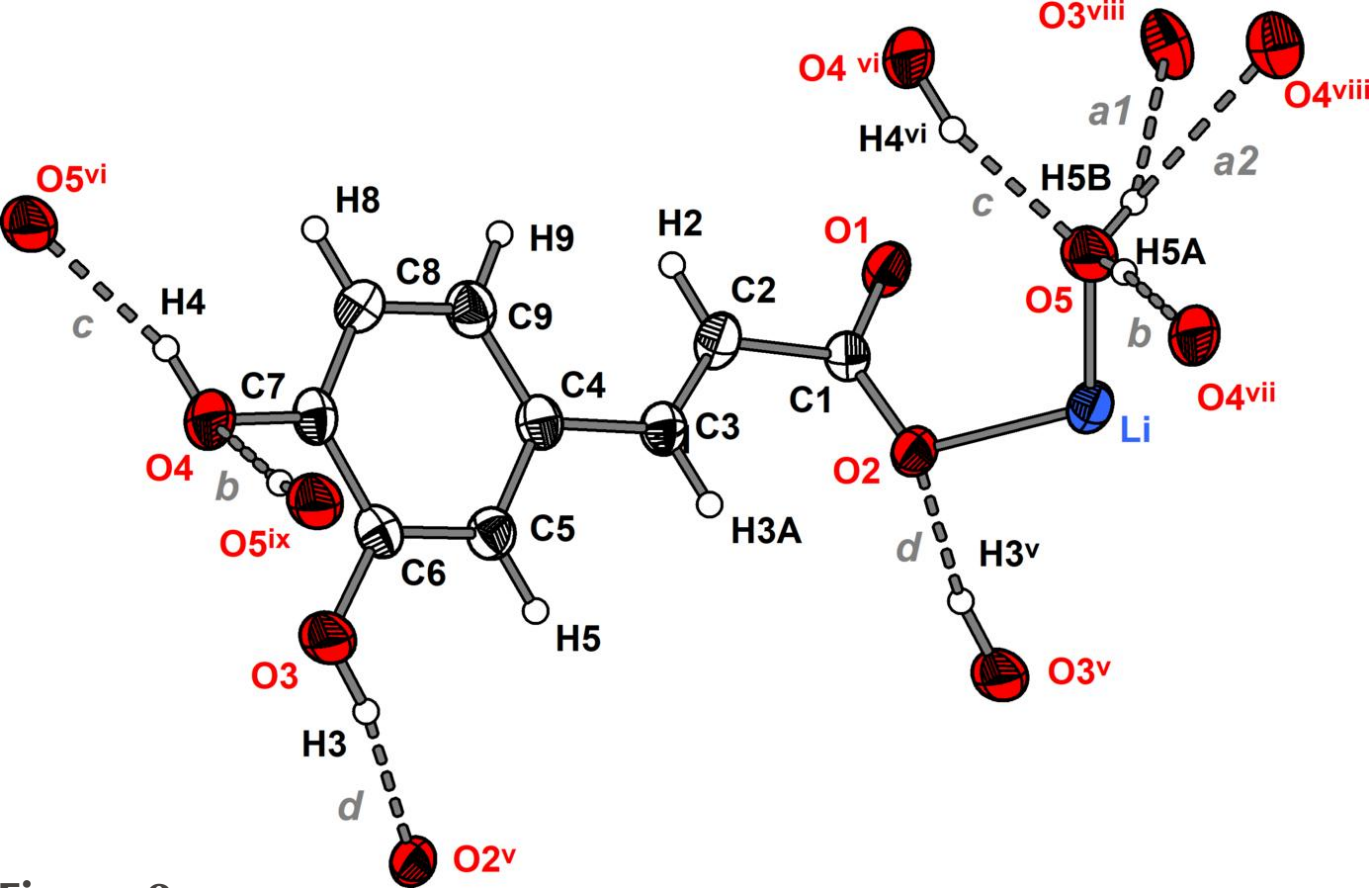
Hydrogen-bonding patterns in **1**. Symmetry codes refer to Table 4 ▸. Additional symmetry codes: (*x*) −*x* + 2, −*y*, −*z*; (xi) *x* + 1, *y*, *z*; (xii) *x -* 2, −*y*, −*z* + 1.

The supra­molecular structure of lithium caffeate hydrate is governed by O—H⋯O hydrogen bonds (Table 4 ▸). Both hydrogen atoms H5*A* and H5*B* of the water mol­ecule are involved in hydrogen bonds to adjacent catechol groups (Fig. 8 ▸). H5*B* is part of a bifurcated hydrogen bond that connects the catechol oxygen atoms O3 and O4 with the water oxygen atom O5 (type *a_1_* and *a_2_* in Fig. 8 ▸). H5*A* forms a hydrogen bond to another neighbouring catechol oxygen atom O4 (type *b*). Moreover, the water oxygen atom O5 acts as an acceptor for the O4—H4 hydroxyl group of a further catechol unit in the surrounding (type *c*). An additional type of hydrogen bond is formed between the catechol group O3—H3 as the donor and the carboxyl­ate oxygen atom O2 as an acceptor (type *d*). The corresponding hydrogen bonds can be considered as medium-strong to weak, with the shortest O⋯O distance found for the *d* type [2.734 (2) Å] hydrogen bond and the largest for the bifurcated *a_1_* type hydrogen bond [3.042 (2) Å].

**Figure 9:**
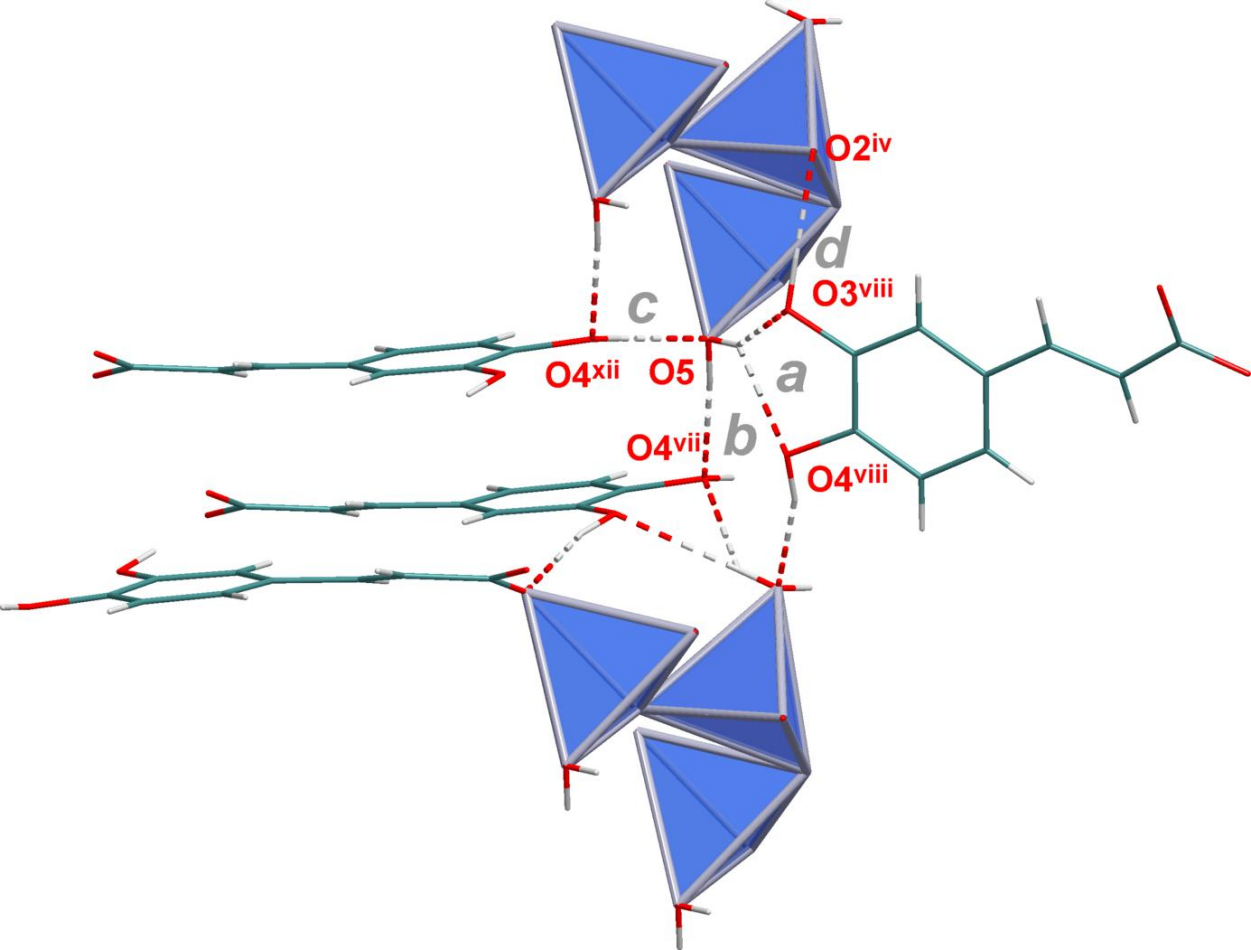
Inter­connection of the LiO_4_ tetra­hedra chains of **1** by hydrogen bonds (dashed lines). Symmetry codes refer to Tables 1 ▸ and 4 ▸. Additional symmetry code: (xii) *x* − 2, −*y*, −*z* + 1.

**Figure 11:**
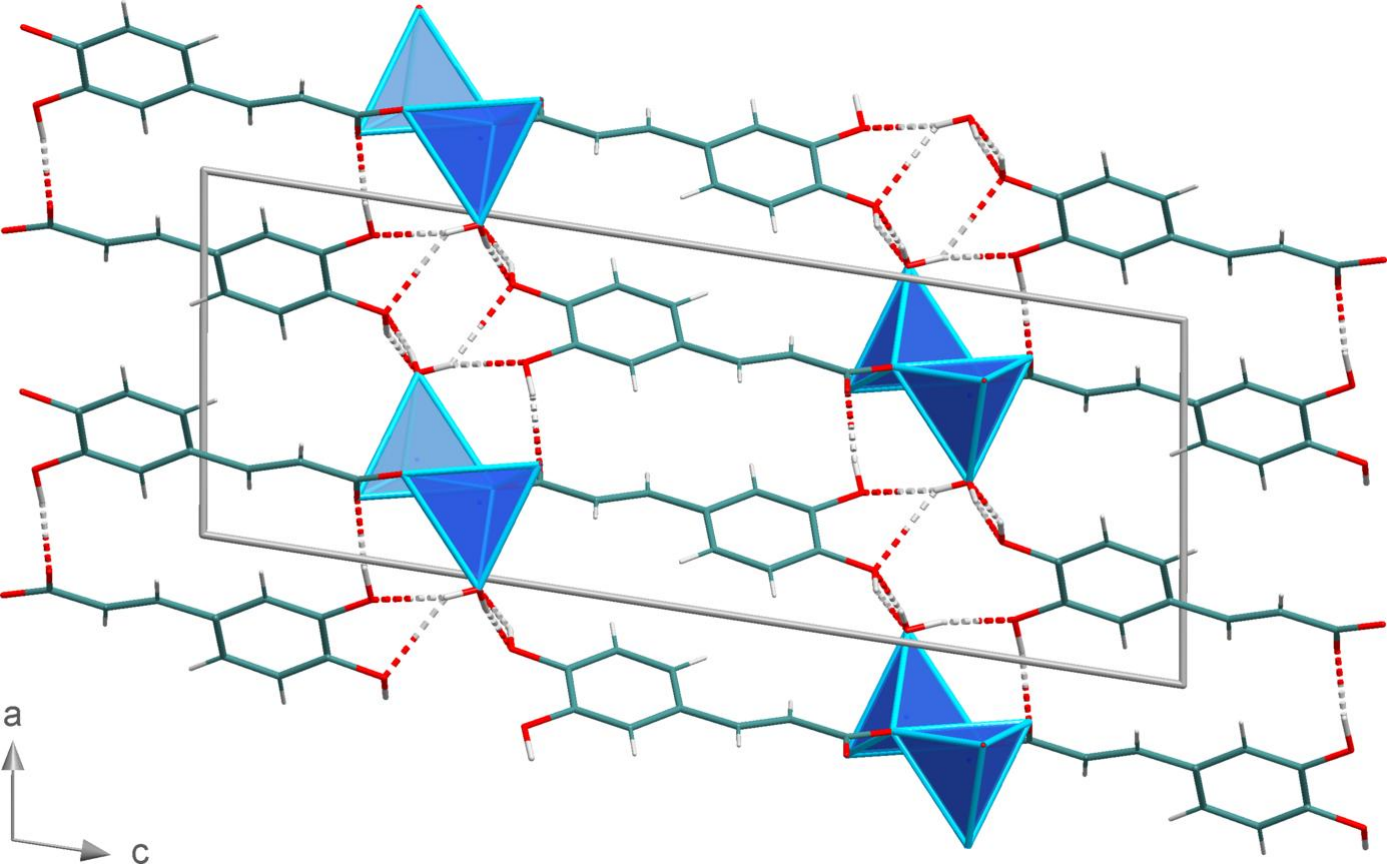
Section of the crystal structure of **1** with the complete hydrogen-bonding network.

Regarding the LiO_4_ tetra­hedra chains, the hydrogen bonds are essential for intra-chain and inter-chain supra­molecular organization. Within a chain, directly adjacent LiO_4_ tetra­hedra are linked pairwise (1,2 connection) by a sequence of hydrogen bonds of the type *a_1_*–*d* starting from O5 (Figs. 9 ▸, 10 ▸). Additionally, there is a *c–b* hydrogen-bonding sequence starting from O5 that links two LiO_4_ tetra­hedra, which are separated by one LiO_4_ unit (1,3 connection). The inter­connection of the LiO_4_ tetra­hedra chains is based on two *a_2_-c* hydrogen-bonding sequences starting from O5 or its centrosymmetric equivalent in the neighbouring chain. This leads to 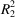 (24) motifs (Fig. 11 ▸).

**Figure 12:**
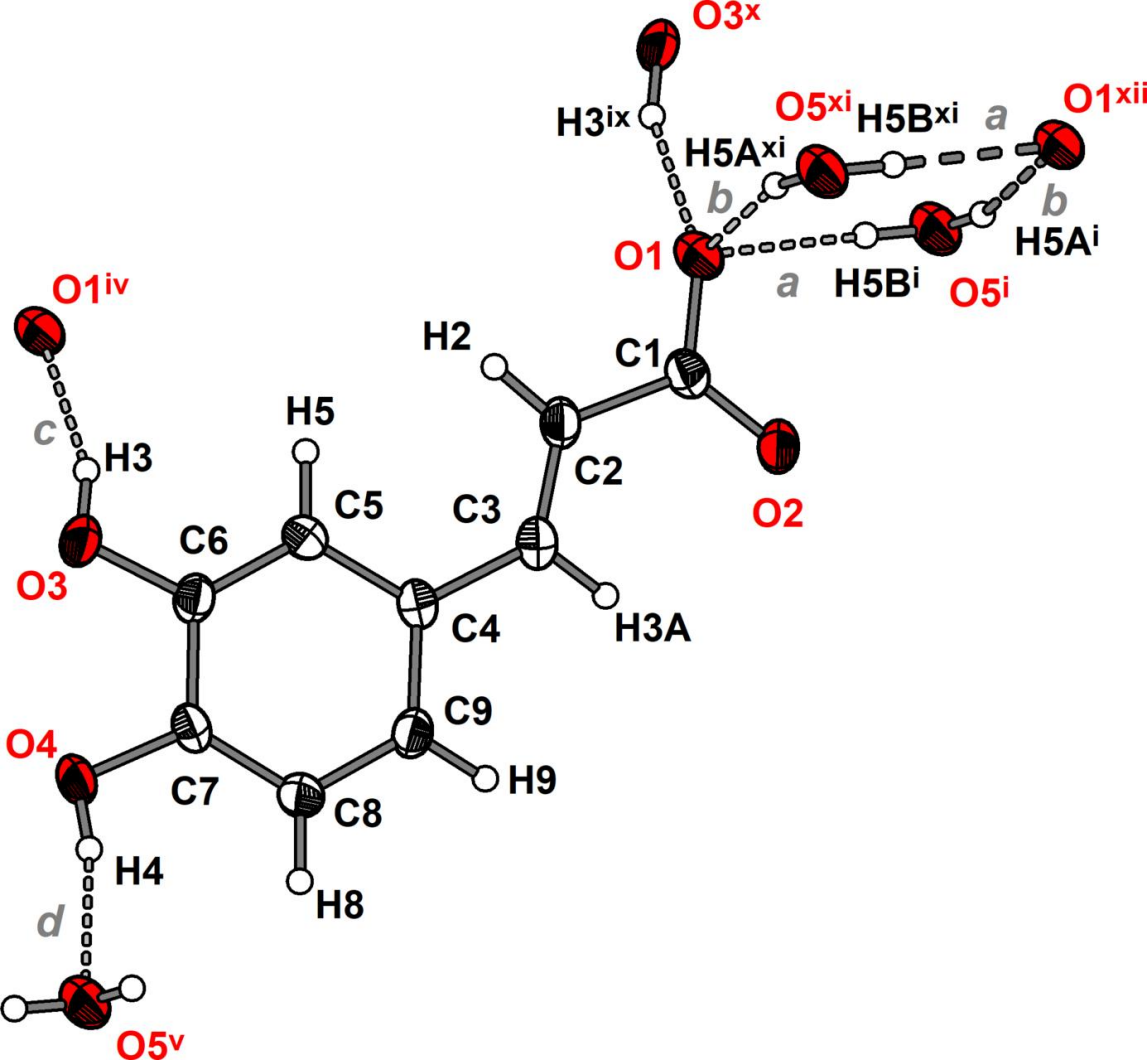
Hydrogen-bonding patterns in the crystal structure of **2**. Symmetry codes refer to Table 5 ▸.

**Figure 13:**
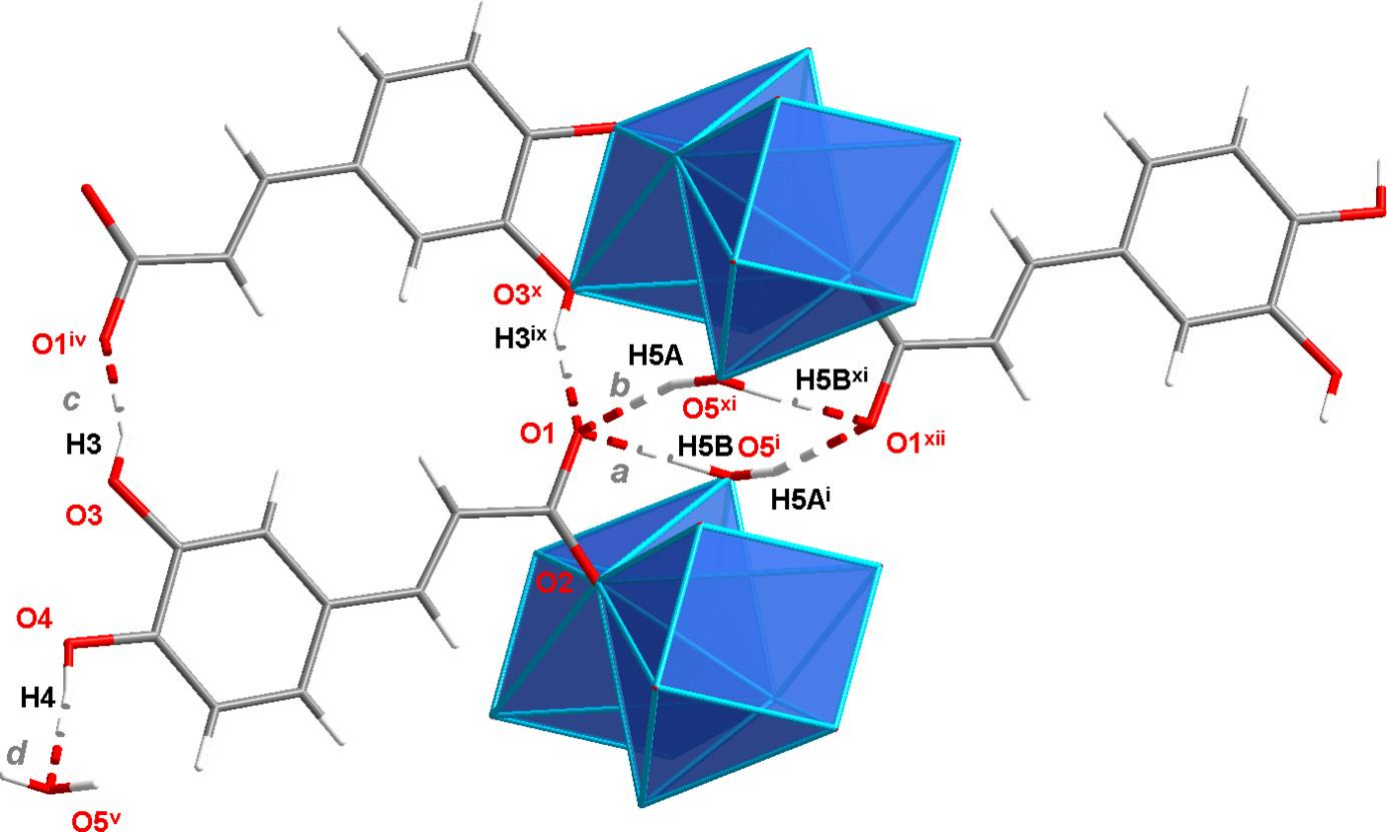
Inter-layer hydrogen bonds in **2.** Symmetry codes refer to Table 5 ▸.

As in the case of **1**, O—H⋯O hydrogen bonds are essential for the supra­molecular organisation within the crystal structure of **2** (Table 5 ▸). The water mol­ecule (H5*A*—O1—H5*B*) participates in two hydrogen bonds (Fig. 12 ▸). The hydrogen bond O5^i^—H5*B* ^i^⋯O1 with the carboxyl­ate oxygen atom as an acceptor (equivalent to O5^xi^—H5*B* ^xi^ ⋯O1^xii^, type *a* in Fig. 12 ▸) acts as an intra-layer linkage, and the hydrogen bond O5^xi^—H5*A* ^xi^—O1 (equivalent to O5^i^—H5*A* ^i^⋯O1^xii^, type *b* in Fig. 12 ▸) connects adjacent layers (Fig. 13 ▸).

**Figure 14:**
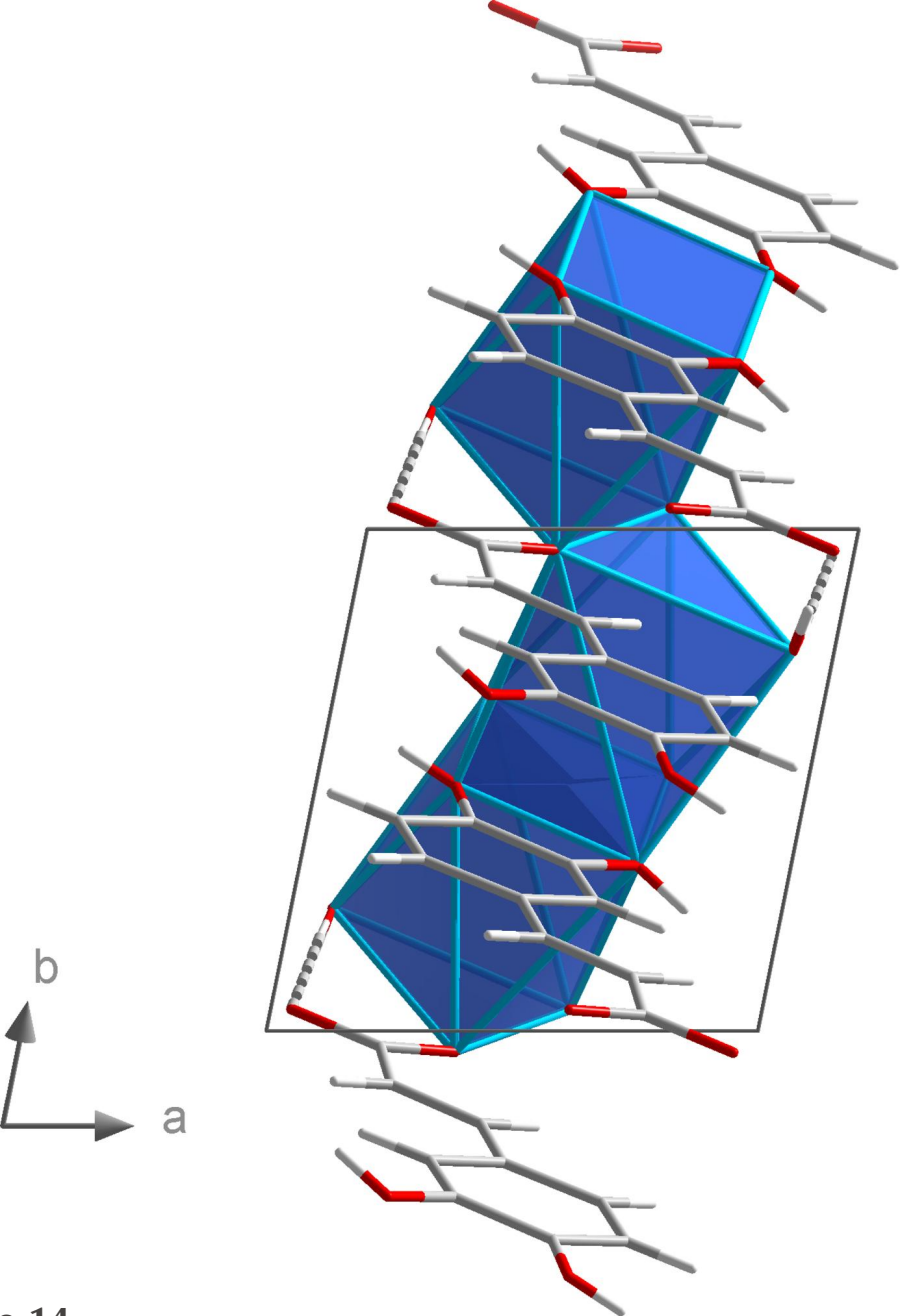
Intra-chain hydrogen bonds in compound **2**.

**Figure 15:**
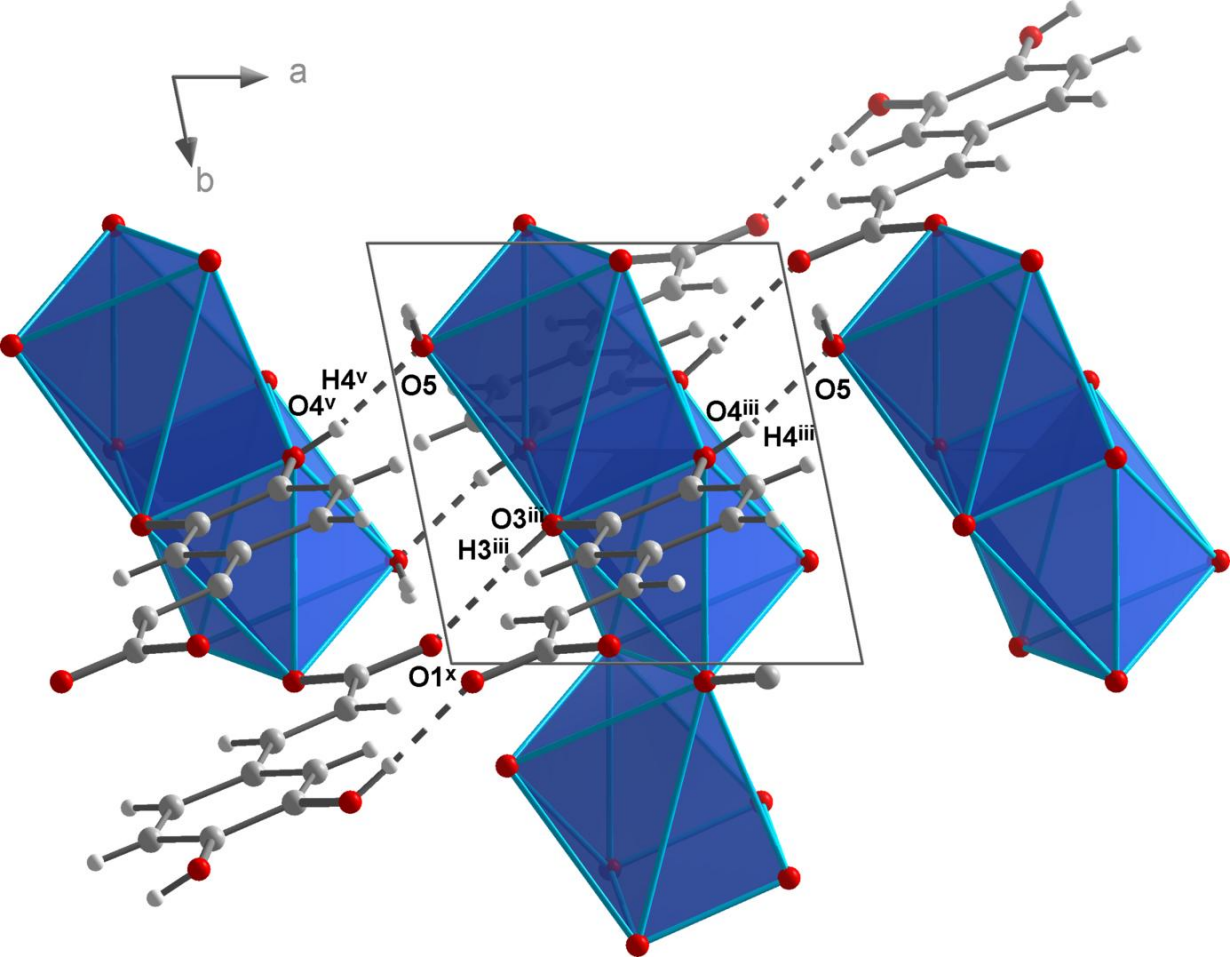
Position of the hydrogen bonds (dashed lines) along the NaO_7_ polyhedral chains in the crystal structure of **2**. Symmetry codes refer to Table 3 ▸. Additional symmetry codes: (x) *x* − 1, *y* + 1, *z*; (xi) *x* + 1, *y*, *z*.

**Figure 16:**
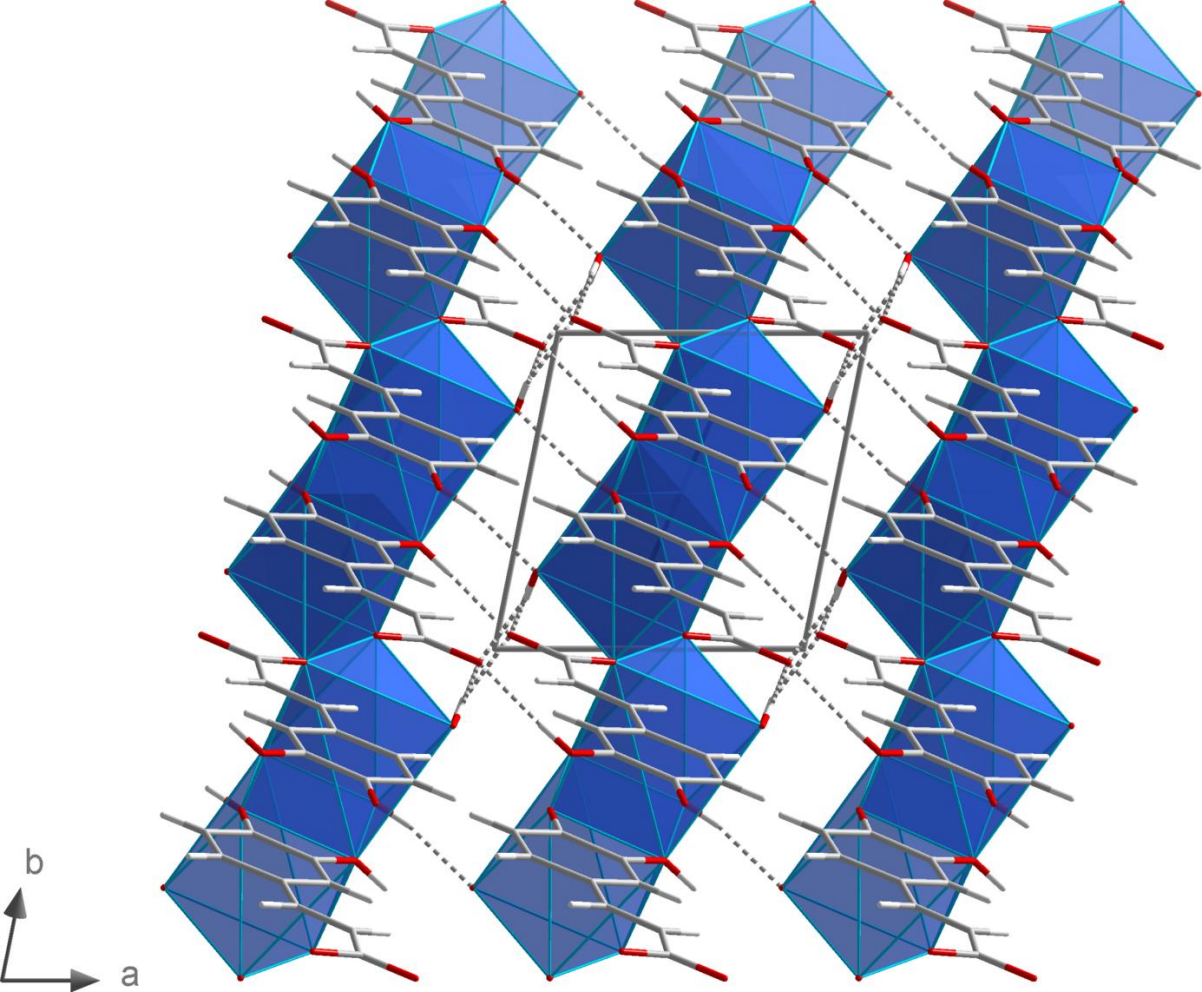
Section of the crystal structure of **2** showing the complete hydrogen-bonding network (dashed lines).

Fig. 14 ▸ represents the position of the intra-chain hydrogen bonds. Furthermore, the catechol groups are involved in hydrogen-bonding inter­actions. The O3—H3 group acts as the donor with respect to the carboxyl­ate oxygen atom O1 of a neighbouring chain (O^iv^ in Fig. 12 ▸, type *c*). This leads to an 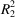 (18) motif between adjacent 3-(3,4-di­hydroxy­phen­yl)-2-propenoate units (Fig. 13 ▸). Finally, the cross-linking of the chains is completed by hydrogen bonds of the type *d* with the O4—H4 group as the donor and the water oxygen atom O5 of a neighbouring chain as acceptor. The position of the different hydrogen-bonding types is displayed in Fig. 15 ▸. Fig. 16 ▸ shows a packing diagram with the complete hydrogen-bonding network of **2**. In direct comparison with **1**, the hydrogen bonds in **2** are significantly stronger, with the closest O⋯O distance being 2.6350 (19) Å (Table 5 ▸).

## 4. Database survey

A search of the Cambridge Structural Database (CSD, Version 2022.3; Groom *et al.*, 2016 ▸) for metal caffeates revealed only the crystal structure of the potassium caffeate/caffeic acid co-crystallization product (CSD code GIFXEA; Lombardo *et al.*, 2012 ▸). Furthermore, there are 16 crystal structures containing caffeic acid as free acid, hydrate or co-crystals with various organic mol­ecules.

## 5. Synthesis and crystallization

2 mmol of the alkali hydroxide (48 mg, LiOH, 80 mg NaOH) and 2 mmol of caffeic acid (360 mg) were dissolved in 5 ml of water to give a clear solution. After slow evaporation the products were isolated as colourless solids in nearly qu­anti­tative yield. Single crystals were obtained by recrystallization from water.

Compound **1**: IR: 1643 *m*, 1616 *w*, 1548 *s*, 1524 *m*, 1441 *w*, 1401 *vs*, 1360 *w*, 1304 *w*, 1249 *vs*, 1195 *w*, 1164 *s*, 1109 *s*, 971 *s*, 859 *s*, 809 *s*, 714 *s*, 602 *w*, 581 *s* cm^−1^.

Compound **2**: IR: 1641 *m*, 1601 *m*, 1519 *s*, 1474 *w*, 1409 *w*, 1369 *s*, 1290 *w*, 1273 *m*, 1250 *s*, 1224 *w*, 1195 *m*, 1012 *w*, 969 *s*, 829 *w*, 873 *w*, 859 *m*, 814 *s*, 739 *m*, 695 *m*, 600 *m*, 564 *s*, 516 *m* cm^−1^.

## 6. Refinement

Crystal data, data collection and structure refinement details are summarized in Table 6 ▸. All carbon-bound hydrogen atoms were positioned geometrically (C—H = 0.94 Å) and refined as riding, with *U*_iso_(H) = 1.2*U*_eq_(C). Hydrogen atoms bound to oxygen were found in difference-Fourier maps. The O—H distances of **2** were restricted to 0.82 Å.

## Supplementary Material

Crystal structure: contains datablock(s) 1, 2, New_Global_Publ_Block. DOI: 10.1107/S2056989024002494/wm5710sup1.cif

Structure factors: contains datablock(s) 1. DOI: 10.1107/S2056989024002494/wm57101sup2.hkl

Structure factors: contains datablock(s) 2. DOI: 10.1107/S2056989024002494/wm57102sup3.hkl

CCDC references: 2340673, 2340672

Additional supporting information:  crystallographic information; 3D view; checkCIF report

## Figures and Tables

**Figure 10:**
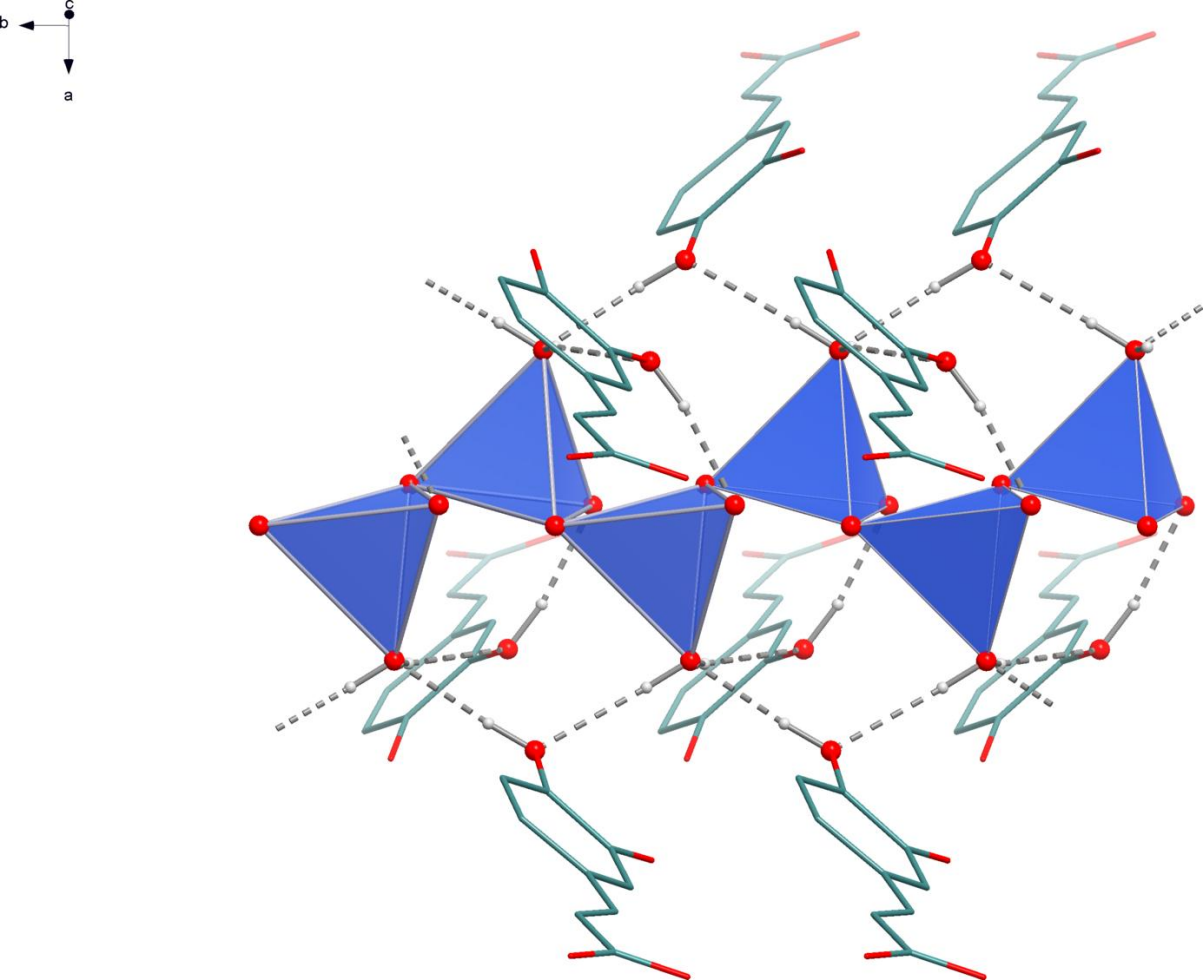
Position of the hydrogen bonds (dashed lines) along the LiO_4_ tetra­hedra chains in the crystal structure of **1**.

**Table 1:** Selected geometric parameters (Å, °) for **1** [Chem scheme1]

C1—C2	1.4855 (18)	C7—O4	1.3781 (16)
C1—O1	1.2669 (16)	Li—O1^i^	1.949 (2)
C1—O2	1.2624 (16)	Li—O1^ii^	1.908 (2)
C2—C3	1.3285 (19)	Li—O2	1.962 (2)
C3—C4	1.4685 (18)	Li—O5	2.005 (3)
C6—O3	1.3757 (17)		
			
O1—C1—C2	117.48 (12)	O1^i^—Li—O5	105.35 (12)
O1^ii^—Li—O1^i^	112.20 (11)	O1^ii^—Li—O5	109.92 (12)
O1^i^—Li—O2	108.08 (12)	O2—Li—O5	110.08 (11)
O1^ii^—Li—O2	111.04 (12)	Li^iii^—O1—Li^iv^	101.15 (8)

Symmetry codes: (i) 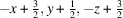 ; (ii) 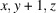 ; (iii) 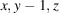 ; (iv) 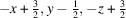 .

**Table 2:** Continuous shape measurement (CShM) values (*SHAPE 2.1*) for the seven-coordinate sodium ion of **2**

Heptagon HP-7	32.482
Hexagonal pyramid HPY-7	20.852
Penta­gonal bipyramid PBPY-7	5.863
Capped octa­hedron COC-7	2.807
Capped trigonal prism CTPR-7	3.593
Johnson penta­gonal bipyramid JPBPY-7	8.482
Johnson elongated triangular pyramid JETPY-7	20.721

**Table 3:** Selected geometric parameters (Å, °) for **2** [Chem scheme1]

C1—C2	1.481 (2)	Na—O2^i^	2.4608 (15)
C1—O2	1.252 (2)	Na—O2	2.3185 (14)
C1—O1	1.282 (2)	Na—O3^ii^	2.7897 (17)
C2—C3	1.327 (2)	Na—O3^iii^	2.7668 (16)
C3—C4	1.463 (2)	Na—O4^iii^	2.5810 (16)
C6—O3	1.3695 (19)	Na—O4^ii^	2.4153 (15)
C7—O4	1.3715 (19)	Na—O5	2.4411 (16)
			
O2—C1—C2	120.37 (14)	O2—Na—O4^ii^	175.93 (5)
O2—Na—O2^i^	84.95 (5)	O2—Na—O4^iii^	90.18 (5)
O2^i^—Na—O3^ii^	83.95 (5)	O2^i^—Na—O4^iii^	145.56 (5)
O2—Na—O3^ii^	118.94 (5)	O2—Na—O5	92.33 (5)
O2—Na—O3^iii^	112.69 (5)	Na—O2—Na^i^	95.05 (5)

Symmetry codes: (i) 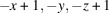 ; (ii) 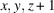 ; (iii) 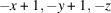 .

**Table 4:** Hydrogen-bond geometry (Å, °) for **1** [Chem scheme1]

*D*—H⋯*A*	*D*—H	H⋯*A*	*D*⋯*A*	*D*—H⋯*A*
O3—H3⋯O2^v^	0.92 (2)	1.83 (2)	2.734 (2)	168 (2)
O4—H4⋯O5^vi^	0.88 (2)	1.94 (2)	2.793 (2)	160.7 (2)
O5—H5*A*⋯O4^vii^	0.85 (3)	2.13 (2)	2.977 (2)	173 (2)
O5—H5*B*⋯O3^viii^	0.80 (2)	2.33 (2)	3.042 (2)	150 (2)
O5—H5*B*⋯O4^viii^	0.80 (2)	2.33 (2)	2.953 (2)	136 (2)

Symmetry codes: (v) 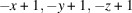 ; (vi) 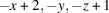 ; (vii) 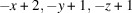 ; (viii) 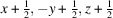 .

**Table 5:** Hydrogen-bond geometry (Å, °) for **2** [Chem scheme1]

*D*—H⋯*A*	*D*—H	H⋯*A*	*D*⋯*A*	*D*—H⋯*A*
O3—H3⋯O1^iv^	0.84 (2)	1.84 (2)	2.6437 (19)	158 (2)
O4—H4⋯O5^v^	0.81 (2)	1.85 (2)	2.6350 (19)	166 (2)
O5—H5*A*⋯O1^vi^	0.83 (2)	2.02 (2)	2.826 (2)	163 (2)
O5—H5*B*⋯O1^i^	0.82 (2)	1.95 (2)	2.7614 (19)	178 (3)

Symmetry codes: (i) 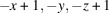 ; (iv) 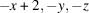 ; (v) 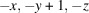 ; (vi) 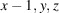 .

**Table 6:** Experimental details

	**1**	**2**
Crystal data		
Chemical formula	[Li(C_9_H_7_O_4_)(H_2_O)]	[Na(C_9_H_7_O_4_)(H_2_O)]
*M*_r_	204.10	220.15
Crystal system, space group	Monoclinic, *P*2_1_/*n*	Triclinic, *P* 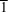
Temperature (K)	220	293
*a*, *b*, *c* (Å)	8.3083 (12), 4.8511 (5), 22.587 (4)	6.3289 (13), 6.8126 (14), 11.253 (2)
α, β, γ (°)	90, 98.572 (18), 90	75.05 (2), 86.39 (2), 78.09 (2)
*V* (Å^3^)	900.2 (2)	458.64 (17)
*Z*	4	2
Radiation type	Mo *K*α	Mo *K*α
μ (mm^−1^)	0.12	0.17
Crystal size (mm)	0.46 × 0.15 × 0.15	0.4 × 0.2 × 0.08

Data collection		
Diffractometer	Stoe *IPDS* 2	Stoe IPDS 2
No. of measured, independent and observed [*I* > 2σ(*I*)] reflections	4973, 1735, 1314	7192, 1798, 1331
*R*_int_	0.027	0.048
(sin θ/λ)_max_ (Å^−1^)	0.619	0.619

Refinement		
*R*[*F* ^2^ > 2σ(*F* ^2^)], *wR*(*F* ^2^), *S*	0.032, 0.079, 1.01	0.035, 0.095, 1.00
No. of reflections	1735	1798
No. of parameters	152	152
No. of restraints	0	4
H-atom treatment	H atoms treated by a mixture of independent and constrained refinement	H atoms treated by a mixture of independent and constrained refinement
Δρ_max_, Δρ_min_ (e Å^−3^)	0.19, −0.15	0.24, −0.22

Computer programs: *X-AREA* (Stoe & Cie, 2016 ▸), *SHELXT* (Sheldrick, 2015*a* ▸), *SHELXL* (Sheldrick, 2015*b* ▸), *DIAMOND* (Brandenburg & Putz, 2019 ▸) and *OLEX2* (Dolomanov *et al.*, 2009 ▸).

## supplementary crystallographic information

### Poly[aqua[µ-3-(3,4-dihydroxyphenyl)propenoato]lithium] (1) Crystal data

**Table:**

[Li(C_9_H_7_O_4_)(H_2_O)]	*F*(000) = 424
*M**_r_* = 204.10	*D*_x_ = 1.506 Mg m^−^^3^
Monoclinic, *P*2_1_/*n*	Mo *K*α radiation, λ = 0.71073 Å
*a* = 8.3083 (12) Å	Cell parameters from 3964 reflections
*b* = 4.8511 (5) Å	θ = 2.5–26.0°
*c* = 22.587 (4) Å	µ = 0.12 mm^−^^1^
β = 98.572 (18)°	*T* = 220 K
*V* = 900.2 (2) Å^3^	Block, colourless
*Z* = 4	0.46 × 0.15 × 0.15 mm

### Poly[aqua[µ-3-(3,4-dihydroxyphenyl)propenoato]lithium] (1) Data collection

**Table:**

Stoe IPDS 2 diffractometer	1314 reflections with *I* > 2σ(*I*)
Radiation source: sealed X-ray tube, 12 x 0.4 mm long-fine focus, Incoatec Iµs	*R*_int_ = 0.027
Plane graphite monochromator	θ_max_ = 26.1°, θ_min_ = 2.5°
Detector resolution: 6.67 pixels mm^-1^	*h* = −10→10
rotation method scans	*k* = −5→5
4973 measured reflections	*l* = −27→27
1735 independent reflections	

### Poly[aqua[µ-3-(3,4-dihydroxyphenyl)propenoato]lithium] (1) Refinement

**Table:**

Refinement on *F*^2^	Primary atom site location: structure-invariant direct methods
Least-squares matrix: full	Hydrogen site location: mixed
*R*[*F*^2^ > 2σ(*F*^2^)] = 0.032	H atoms treated by a mixture of independent and constrained refinement
*wR*(*F*^2^) = 0.079	*w* = 1/[σ^2^(*F*_o_^2^) + (0.047*P*)^2^ + 0.0259*P*] where *P* = (*F*_o_^2^ + 2*F*_c_^2^)/3
*S* = 1.01	(Δ/σ)_max_ = 0.001
1735 reflections	Δρ_max_ = 0.19 e Å^−^^3^
152 parameters	Δρ_min_ = −0.15 e Å^−^^3^
0 restraints	

### Poly[aqua[µ-3-(3,4-dihydroxyphenyl)propenoato]lithium] (1) Special details

**Table:**

Geometry. All esds (except the esd in the dihedral angle between two l.s. planes) are estimated using the full covariance matrix. The cell esds are taken into account individually in the estimation of esds in distances, angles and torsion angles; correlations between esds in cell parameters are only used when they are defined by crystal symmetry. An approximate (isotropic) treatment of cell esds is used for estimating esds involving l.s. planes.

### Poly[aqua[µ-3-(3,4-dihydroxyphenyl)propenoato]lithium] (1) Fractional atomic coordinates and isotropic or equivalent isotropic displacement parameters (Å^2^)

**Table:**

	*x*	*y*	*z*	*U*_iso_*/*U*_eq_	
C1	0.71575 (16)	0.1874 (3)	0.65653 (5)	0.0232 (3)	
C2	0.74035 (18)	0.0573 (3)	0.59901 (6)	0.0297 (3)	
H2	0.787147	−0.119367	0.599929	0.036*	
C3	0.69907 (16)	0.1798 (3)	0.54634 (6)	0.0274 (3)	
H3A	0.647720	0.352195	0.547117	0.033*	
C4	0.72381 (16)	0.0791 (3)	0.48705 (6)	0.0265 (3)	
C5	0.64958 (16)	0.2210 (3)	0.43618 (6)	0.0279 (3)	
H5	0.579486	0.369071	0.440744	0.034*	
C6	0.67740 (16)	0.1475 (3)	0.37929 (6)	0.0272 (3)	
C7	0.78474 (16)	−0.0658 (3)	0.37246 (6)	0.0271 (3)	
C8	0.85657 (17)	−0.2130 (3)	0.42205 (6)	0.0307 (3)	
H8	0.926536	−0.360889	0.417203	0.037*	
C9	0.82555 (18)	−0.1427 (3)	0.47885 (6)	0.0315 (3)	
H9	0.873489	−0.245454	0.512161	0.038*	
Li	0.7956 (3)	0.6632 (5)	0.71799 (10)	0.0283 (5)	
O1	0.75014 (12)	0.0450 (2)	0.70375 (4)	0.0297 (3)	
O2	0.66671 (12)	0.43357 (19)	0.65694 (4)	0.0294 (3)	
O3	0.60783 (14)	0.2806 (2)	0.32801 (4)	0.0376 (3)	
H3	0.525 (3)	0.395 (4)	0.3355 (9)	0.060 (6)*	
O4	0.81610 (14)	−0.1140 (3)	0.31514 (4)	0.0362 (3)	
H4	0.869 (3)	−0.271 (5)	0.3134 (9)	0.068 (7)*	
O5	1.03368 (14)	0.5915 (3)	0.71894 (5)	0.0361 (3)	
H5A	1.082 (3)	0.740 (6)	0.7122 (11)	0.092 (9)*	
H5B	1.070 (3)	0.548 (5)	0.7522 (11)	0.070 (7)*	

### Poly[aqua[µ-3-(3,4-dihydroxyphenyl)propenoato]lithium] (1) Atomic displacement parameters (Å^2^)

**Table:**

	*U*^11^	*U*^22^	*U*^33^	*U*^12^	*U*^13^	*U*^23^
C1	0.0269 (7)	0.0219 (7)	0.0211 (7)	−0.0027 (6)	0.0043 (5)	0.0003 (5)
C2	0.0412 (8)	0.0232 (7)	0.0258 (7)	−0.0006 (6)	0.0089 (6)	−0.0027 (6)
C3	0.0281 (7)	0.0301 (8)	0.0245 (7)	−0.0019 (6)	0.0056 (5)	−0.0028 (6)
C4	0.0272 (7)	0.0298 (8)	0.0232 (7)	−0.0049 (6)	0.0056 (5)	−0.0021 (6)
C5	0.0265 (7)	0.0327 (8)	0.0255 (7)	0.0004 (6)	0.0068 (5)	−0.0031 (6)
C6	0.0268 (7)	0.0327 (8)	0.0217 (7)	−0.0033 (6)	0.0026 (5)	−0.0009 (5)
C7	0.0276 (7)	0.0323 (8)	0.0222 (7)	−0.0048 (6)	0.0063 (5)	−0.0052 (6)
C8	0.0329 (8)	0.0283 (8)	0.0314 (7)	0.0030 (7)	0.0061 (6)	−0.0039 (6)
C9	0.0377 (8)	0.0315 (8)	0.0249 (7)	−0.0022 (7)	0.0034 (6)	0.0005 (6)
Li	0.0389 (13)	0.0229 (11)	0.0239 (11)	0.0025 (10)	0.0075 (9)	−0.0009 (9)
O1	0.0468 (6)	0.0226 (5)	0.0208 (5)	0.0037 (4)	0.0087 (4)	0.0015 (4)
O2	0.0406 (6)	0.0227 (5)	0.0242 (5)	0.0047 (5)	0.0019 (4)	−0.0011 (4)
O3	0.0420 (6)	0.0486 (7)	0.0220 (5)	0.0132 (6)	0.0038 (4)	0.0016 (5)
O4	0.0455 (6)	0.0419 (7)	0.0229 (5)	0.0066 (6)	0.0106 (4)	−0.0046 (5)
O5	0.0378 (6)	0.0444 (7)	0.0261 (6)	0.0060 (6)	0.0049 (5)	0.0005 (5)

### Poly[aqua[µ-3-(3,4-dihydroxyphenyl)propenoato]lithium] (1) Geometric parameters (Å, º)

**Table:**

C1—C2	1.4855 (18)	C7—O4	1.3781 (16)
C1—O1	1.2669 (16)	C8—H8	0.9400
C1—O2	1.2624 (16)	C8—C9	1.388 (2)
C2—H2	0.9400	C9—H9	0.9400
C2—C3	1.3285 (19)	Li—Li^i^	2.979 (3)
C3—H3A	0.9400	Li—Li^i^	2.979 (3)
C3—C4	1.4685 (18)	Li—O1^ii^	1.949 (2)
C4—C5	1.401 (2)	Li—O1^iii^	1.908 (2)
C4—C9	1.398 (2)	Li—O2	1.962 (2)
C5—H5	0.9400	Li—O5	2.005 (3)
C5—C6	1.3857 (18)	O3—H3	0.92 (2)
C6—C7	1.390 (2)	O4—H4	0.88 (3)
C6—O3	1.3757 (17)	O5—H5A	0.85 (3)
C7—C8	1.386 (2)	O5—H5B	0.79 (3)
			
O1—C1—C2	117.48 (12)	C8—C9—H9	119.6
O2—C1—C2	119.66 (12)	Li^i^—Li—Li^ii^	109.00 (13)
O2—C1—O1	122.83 (12)	O1^iii^—Li—Li^ii^	39.93 (3)
C1—C2—H2	118.6	O1^iii^—Li—Li^i^	144.46 (14)
C3—C2—C1	122.84 (13)	O1^ii^—Li—Li^i^	38.92 (9)
C3—C2—H2	118.6	O1^ii^—Li—Li^ii^	72.61 (11)
C2—C3—H3A	116.0	O1^iii^—Li—O1^ii^	112.20 (11)
C2—C3—C4	127.96 (14)	O1^ii^—Li—O2	108.08 (12)
C4—C3—H3A	116.0	O1^iii^—Li—O2	111.04 (12)
C5—C4—C3	118.68 (13)	O1^ii^—Li—O5	105.35 (12)
C9—C4—C3	123.15 (12)	O1^iii^—Li—O5	109.92 (12)
C9—C4—C5	118.07 (12)	O2—Li—Li^i^	74.16 (8)
C4—C5—H5	119.3	O2—Li—Li^ii^	130.66 (15)
C6—C5—C4	121.35 (14)	O2—Li—O5	110.08 (11)
C6—C5—H5	119.3	O5—Li—Li^i^	100.11 (11)
C5—C6—C7	119.53 (12)	O5—Li—Li^ii^	117.27 (13)
O3—C6—C5	123.57 (13)	C1—O1—Li^iv^	133.25 (11)
O3—C6—C7	116.87 (11)	C1—O1—Li^i^	123.58 (11)
C8—C7—C6	120.01 (12)	Li^iv^—O1—Li^i^	101.15 (8)
O4—C7—C6	116.41 (12)	C1—O2—Li	113.53 (11)
O4—C7—C8	123.56 (13)	C6—O3—H3	111.2 (12)
C7—C8—H8	119.9	C7—O4—H4	110.7 (14)
C7—C8—C9	120.23 (13)	Li—O5—H5A	110.0 (18)
C9—C8—H8	119.9	Li—O5—H5B	106.7 (16)
C4—C9—H9	119.6	H5A—O5—H5B	106 (2)
C8—C9—C4	120.72 (13)		
			
C1—C2—C3—C4	176.94 (13)	C5—C6—C7—O4	−175.22 (12)
C2—C1—O1—Li^iv^	−12.6 (2)	C6—C7—C8—C9	−2.0 (2)
C2—C1—O1—Li^i^	−173.12 (12)	C7—C8—C9—C4	−1.0 (2)
C2—C1—O2—Li	−131.35 (13)	C9—C4—C5—C6	−0.9 (2)
C2—C3—C4—C5	170.59 (14)	O1—C1—C2—C3	175.69 (13)
C2—C3—C4—C9	−13.2 (2)	O1—C1—O2—Li	46.58 (17)
C3—C4—C5—C6	175.49 (13)	O2—C1—C2—C3	−6.3 (2)
C3—C4—C9—C8	−173.87 (13)	O2—C1—O1—Li^iv^	169.48 (14)
C4—C5—C6—C7	−1.9 (2)	O2—C1—O1—Li^i^	8.9 (2)
C4—C5—C6—O3	−179.85 (13)	O3—C6—C7—C8	−178.57 (13)
C5—C4—C9—C8	2.4 (2)	O3—C6—C7—O4	2.84 (18)
C5—C6—C7—C8	3.4 (2)	O4—C7—C8—C9	176.53 (13)

Symmetry codes: (i) −*x*+3/2, *y*−1/2, −*z*+3/2; (ii) −*x*+3/2, *y*+1/2, −*z*+3/2; (iii) *x*, *y*+1, *z*; (iv) *x*, *y*−1, *z*.

### Poly[aqua[µ-3-(3,4-dihydroxyphenyl)propenoato]lithium] (1) Hydrogen-bond geometry (Å, º)

**Table:**

*D*—H···*A*	*D*—H	H···*A*	*D*···*A*	*D*—H···*A*
O3—H3···O2^v^	0.92 (2)	1.83 (2)	2.734 (2)	168 (2)
O4—H4···O5^vi^	0.88 (2)	1.94 (2)	2.793 (2)	160.7 (2)
O5—H5*A*···O4^vii^	0.85 (3)	2.13 (2)	2.977 (2)	173 (2)
O5—H5*B*···O3^viii^	0.80 (2)	2.33 (2)	3.042 (2)	150 (2)
O5—H5*B*···O4^viii^	0.80 (2)	2.33 (2)	2.953 (2)	136 (2)

Symmetry codes: (v) −*x*+1, −*y*+1, −*z*+1; (vi) −*x*+2, −*y*, −*z*+1; (vii) −*x*+2, −*y*+1, −*z*+1; (viii) *x*+1/2, −*y*+1/2, *z*+1/2.

### Poly[aqua[µ-3-(3,4-dihydroxyphenyl)propenoato]sodium] (2) Crystal data

**Table:**

[Na(C_9_H_7_O_4_)(H_2_O)]	*Z* = 2
*M**_r_* = 220.15	*F*(000) = 228
Triclinic, *P*1	*D*_x_ = 1.594 Mg m^−^^3^
*a* = 6.3289 (13) Å	Mo *K*α radiation, λ = 0.71073 Å
*b* = 6.8126 (14) Å	Cell parameters from 2529 reflections
*c* = 11.253 (2) Å	θ = 3.1–26.0°
α = 75.05 (2)°	µ = 0.17 mm^−^^1^
β = 86.39 (2)°	*T* = 293 K
γ = 78.09 (2)°	Plate, colourless
*V* = 458.64 (17) Å^3^	0.4 × 0.2 × 0.08 mm

### Poly[aqua[µ-3-(3,4-dihydroxyphenyl)propenoato]sodium] (2) Data collection

**Table:**

Stoe IPDS 2 diffractometer	1331 reflections with *I* > 2σ(*I*)
Radiation source: sealed X-ray tube, 12 x 0.4 mm long-fine focus, Incoatec Iµs	*R*_int_ = 0.048
Plane graphite monochromator	θ_max_ = 26.1°, θ_min_ = 3.2°
Detector resolution: 6.67 pixels mm^-1^	*h* = −7→7
rotation method scans	*k* = −8→8
7192 measured reflections	*l* = −13→13
1798 independent reflections	

### Poly[aqua[µ-3-(3,4-dihydroxyphenyl)propenoato]sodium] (2) Refinement

**Table:**

Refinement on *F*^2^	Primary atom site location: dual
Least-squares matrix: full	Hydrogen site location: mixed
*R*[*F*^2^ > 2σ(*F*^2^)] = 0.035	H atoms treated by a mixture of independent and constrained refinement
*wR*(*F*^2^) = 0.095	*w* = 1/[σ^2^(*F*_o_^2^) + (0.0615*P*)^2^] where *P* = (*F*_o_^2^ + 2*F*_c_^2^)/3
*S* = 1.00	(Δ/σ)_max_ < 0.001
1798 reflections	Δρ_max_ = 0.24 e Å^−^^3^
152 parameters	Δρ_min_ = −0.22 e Å^−^^3^
4 restraints	

### Poly[aqua[µ-3-(3,4-dihydroxyphenyl)propenoato]sodium] (2) Special details

**Table:**

Geometry. All esds (except the esd in the dihedral angle between two l.s. planes) are estimated using the full covariance matrix. The cell esds are taken into account individually in the estimation of esds in distances, angles and torsion angles; correlations between esds in cell parameters are only used when they are defined by crystal symmetry. An approximate (isotropic) treatment of cell esds is used for estimating esds involving l.s. planes.

### Poly[aqua[µ-3-(3,4-dihydroxyphenyl)propenoato]sodium] (2) Fractional atomic coordinates and isotropic or equivalent isotropic displacement parameters (Å^2^)

**Table:**

	*x*	*y*	*z*	*U*_iso_*/*U*_eq_	
C1	0.7621 (3)	0.0311 (2)	0.28917 (15)	0.0254 (4)	
C2	0.7226 (3)	0.1072 (2)	0.15528 (14)	0.0264 (4)	
H2	0.8399	0.1004	0.1013	0.032*	
C3	0.5262 (3)	0.1847 (2)	0.10941 (15)	0.0255 (4)	
H3A	0.4138	0.1903	0.1666	0.031*	
C4	0.4642 (3)	0.2624 (2)	−0.01989 (14)	0.0227 (3)	
C5	0.6151 (3)	0.2576 (2)	−0.11683 (15)	0.0251 (4)	
H5	0.7604	0.2042	−0.0994	0.030*	
C6	0.5499 (3)	0.3310 (2)	−0.23713 (14)	0.0251 (4)	
C7	0.3322 (3)	0.4163 (2)	−0.26497 (14)	0.0228 (3)	
C8	0.1815 (3)	0.4196 (2)	−0.17098 (14)	0.0259 (4)	
H8	0.0363	0.4734	−0.1888	0.031*	
C9	0.2482 (3)	0.3420 (2)	−0.04956 (14)	0.0260 (4)	
H9	0.1458	0.3434	0.0134	0.031*	
Na	0.44737 (11)	0.27100 (10)	0.47948 (6)	0.0348 (2)	
O1	0.95808 (19)	−0.0433 (2)	0.32325 (11)	0.0363 (3)	
O2	0.60826 (19)	0.04183 (18)	0.36431 (10)	0.0328 (3)	
O3	0.6857 (2)	0.3298 (2)	−0.33673 (11)	0.0387 (3)	
H3	0.800 (3)	0.243 (4)	−0.314 (2)	0.070 (8)*	
O4	0.2845 (2)	0.48950 (18)	−0.38767 (10)	0.0304 (3)	
H4	0.164 (3)	0.558 (3)	−0.391 (2)	0.053 (7)*	
O5	0.0849 (2)	0.2438 (2)	0.43233 (13)	0.0345 (3)	
H5A	0.070 (4)	0.163 (3)	0.391 (2)	0.061 (8)*	
H5B	0.070 (4)	0.187 (4)	0.5048 (17)	0.059 (7)*	

### Poly[aqua[µ-3-(3,4-dihydroxyphenyl)propenoato]sodium] (2) Atomic displacement parameters (Å^2^)

**Table:**

	*U*^11^	*U*^22^	*U*^33^	*U*^12^	*U*^13^	*U*^23^
C1	0.0297 (9)	0.0243 (8)	0.0217 (8)	−0.0013 (6)	−0.0040 (6)	−0.0074 (6)
C2	0.0302 (9)	0.0310 (8)	0.0179 (8)	−0.0074 (7)	0.0019 (6)	−0.0054 (6)
C3	0.0320 (9)	0.0251 (8)	0.0190 (8)	−0.0054 (6)	0.0016 (6)	−0.0054 (6)
C4	0.0291 (8)	0.0213 (7)	0.0177 (8)	−0.0046 (6)	−0.0009 (6)	−0.0048 (6)
C5	0.0235 (8)	0.0271 (8)	0.0220 (8)	−0.0018 (6)	−0.0030 (6)	−0.0031 (6)
C6	0.0274 (8)	0.0261 (8)	0.0197 (8)	−0.0039 (6)	0.0036 (6)	−0.0042 (6)
C7	0.0293 (9)	0.0215 (7)	0.0164 (8)	−0.0023 (6)	−0.0033 (6)	−0.0042 (6)
C8	0.0238 (8)	0.0268 (8)	0.0240 (9)	0.0009 (6)	−0.0024 (6)	−0.0050 (6)
C9	0.0278 (8)	0.0287 (8)	0.0199 (8)	−0.0025 (6)	0.0035 (6)	−0.0066 (6)
Na	0.0381 (4)	0.0366 (4)	0.0316 (4)	−0.0084 (3)	0.0087 (3)	−0.0132 (3)
O1	0.0302 (7)	0.0487 (7)	0.0270 (7)	0.0046 (5)	−0.0078 (5)	−0.0123 (5)
O2	0.0343 (7)	0.0399 (7)	0.0195 (6)	0.0017 (5)	0.0019 (5)	−0.0068 (5)
O3	0.0304 (7)	0.0546 (8)	0.0191 (6)	0.0056 (6)	0.0051 (5)	−0.0004 (5)
O4	0.0323 (7)	0.0360 (6)	0.0165 (6)	0.0050 (5)	−0.0046 (5)	−0.0033 (5)
O5	0.0356 (7)	0.0375 (7)	0.0262 (7)	−0.0005 (5)	−0.0078 (5)	−0.0039 (6)

### Poly[aqua[µ-3-(3,4-dihydroxyphenyl)propenoato]sodium] (2) Geometric parameters (Å, º)

**Table:**

C1—C2	1.481 (2)	Na—Na^ii^	3.4689 (15)
C1—O2	1.252 (2)	Na—O2^i^	2.4608 (15)
C1—O1	1.282 (2)	Na—O2	2.3185 (14)
C2—H2	0.9300	Na—O3^iii^	2.7897 (17)
C2—C3	1.327 (2)	Na—O3^iv^	2.7668 (16)
C3—H3A	0.9300	Na—O4^iv^	2.5810 (16)
C3—C4	1.463 (2)	Na—O4^iii^	2.4153 (15)
C4—C5	1.406 (2)	Na—O5	2.4411 (16)
C4—C9	1.390 (2)	Na—H5B	2.55 (2)
C5—H5	0.9300	O2—Na^i^	2.4608 (15)
C5—C6	1.374 (2)	O3—Na^v^	2.7897 (17)
C6—C7	1.401 (2)	O3—Na^iv^	2.7669 (16)
C6—O3	1.3695 (19)	O3—H3	0.841 (17)
C7—C8	1.380 (2)	O4—Na^iv^	2.5810 (16)
C7—O4	1.3715 (19)	O4—Na^v^	2.4153 (15)
C8—H8	0.9300	O4—H4	0.803 (16)
C8—C9	1.390 (2)	O5—H5A	0.827 (17)
C9—H9	0.9300	O5—H5B	0.816 (17)
Na—Na^i^	3.5262 (15)		
			
O1—C1—C2	117.18 (14)	O3^iii^—Na—Na^ii^	51.08 (4)
O2—C1—C2	120.37 (14)	O3^iv^—Na—Na^i^	151.40 (5)
O2—C1—O1	122.45 (15)	O3^iii^—Na—Na^i^	104.16 (5)
C1—C2—H2	118.8	O3^iv^—Na—Na^ii^	51.67 (4)
C3—C2—C1	122.43 (15)	O3^iv^—Na—O3^iii^	102.74 (4)
C3—C2—H2	118.8	O3^iv^—Na—H5B	94.7 (5)
C2—C3—H3A	116.0	O3^iii^—Na—H5B	126.0 (4)
C2—C3—C4	128.09 (15)	O4^iii^—Na—Na^i^	132.15 (5)
C4—C3—H3A	116.0	O4^iii^—Na—Na^ii^	48.03 (4)
C5—C4—C3	122.50 (15)	O4^iv^—Na—Na^ii^	44.09 (3)
C9—C4—C3	119.45 (14)	O4^iv^—Na—Na^i^	125.28 (5)
C9—C4—C5	118.04 (14)	O4^iii^—Na—O2^i^	91.26 (5)
C4—C5—H5	119.7	O4^iv^—Na—O3^iv^	57.67 (4)
C6—C5—C4	120.66 (15)	O4^iii^—Na—O3^iv^	71.38 (4)
C6—C5—H5	119.7	O4^iv^—Na—O3^iii^	68.74 (4)
C5—C6—C7	120.37 (15)	O4^iii^—Na—O3^iii^	59.01 (4)
O3—C6—C5	124.36 (15)	O4^iii^—Na—O4^iv^	92.12 (5)
O3—C6—C7	115.27 (14)	O4^iii^—Na—O5	88.31 (5)
C8—C7—C6	119.71 (14)	O4^iv^—Na—H5B	152.3 (5)
O4—C7—C6	115.86 (14)	O4^iii^—Na—H5B	80.0 (5)
O4—C7—C8	124.43 (14)	O5—Na—Na^i^	83.14 (5)
C7—C8—H8	120.2	O5—Na—Na^ii^	121.25 (5)
C7—C8—C9	119.57 (14)	O5—Na—O2^i^	77.91 (5)
C9—C8—H8	120.2	O5—Na—O3^iv^	81.58 (5)
C4—C9—H9	119.2	O5—Na—O3^iii^	142.21 (5)
C8—C9—C4	121.62 (15)	O5—Na—O4^iv^	136.45 (5)
C8—C9—H9	119.2	O5—Na—H5B	18.6 (4)
Na^ii^—Na—Na^i^	153.76 (5)	C1—O2—Na	136.46 (11)
Na^ii^—Na—H5B	122.5 (6)	C1—O2—Na^i^	119.95 (10)
Na^i^—Na—H5B	77.2 (5)	Na—O2—Na^i^	95.05 (5)
O2—Na—Na^i^	44.04 (4)	C6—O3—Na^v^	106.29 (10)
O2^i^—Na—Na^i^	40.91 (3)	C6—O3—Na^iv^	101.08 (9)
O2—Na—Na^ii^	134.17 (5)	C6—O3—H3	108.4 (19)
O2^i^—Na—Na^ii^	128.97 (5)	Na^iv^—O3—Na^v^	77.26 (4)
O2—Na—O2^i^	84.95 (5)	Na^iv^—O3—H3	140.1 (19)
O2^i^—Na—O3^iii^	83.95 (5)	Na^v^—O3—H3	117.7 (19)
O2—Na—O3^iii^	118.94 (5)	C7—O4—Na^v^	116.66 (9)
O2—Na—O3^iv^	112.69 (5)	C7—O4—Na^iv^	105.73 (10)
O2^i^—Na—O3^iv^	153.47 (5)	C7—O4—H4	105.9 (17)
O2—Na—O4^iii^	175.93 (5)	Na^v^—O4—Na^iv^	87.88 (5)
O2—Na—O4^iv^	90.18 (5)	Na^v^—O4—H4	127.7 (17)
O2^i^—Na—O4^iv^	145.56 (5)	Na^iv^—O4—H4	109.1 (17)
O2—Na—O5	92.33 (5)	Na—O5—H5A	119.7 (18)
O2—Na—H5B	99.4 (5)	Na—O5—H5B	88.4 (18)
O2^i^—Na—H5B	61.8 (5)	H5A—O5—H5B	108 (2)
			
C1—C2—C3—C4	−179.15 (14)	C6—C7—O4—Na^iv^	−50.68 (14)
C2—C1—O2—Na	−94.99 (19)	C6—C7—O4—Na^v^	44.96 (17)
C2—C1—O2—Na^i^	126.10 (13)	C7—C6—O3—Na^iv^	46.19 (15)
C2—C3—C4—C5	2.1 (3)	C7—C6—O3—Na^v^	−33.62 (16)
C2—C3—C4—C9	−178.71 (16)	C7—C8—C9—C4	0.6 (2)
C3—C4—C5—C6	179.58 (14)	C8—C7—O4—Na^v^	−134.11 (14)
C3—C4—C9—C8	179.34 (14)	C8—C7—O4—Na^iv^	130.25 (14)
C4—C5—C6—C7	1.5 (2)	C9—C4—C5—C6	0.4 (2)
C4—C5—C6—O3	−179.20 (15)	O1—C1—C2—C3	179.07 (15)
C5—C4—C9—C8	−1.5 (2)	O1—C1—O2—Na^i^	−54.4 (2)
C5—C6—C7—C8	−2.3 (2)	O1—C1—O2—Na	84.5 (2)
C5—C6—C7—O4	178.54 (14)	O2—C1—C2—C3	−1.4 (2)
C5—C6—O3—Na^iv^	−133.17 (15)	O3—C6—C7—C8	178.27 (15)
C5—C6—O3—Na^v^	147.02 (14)	O3—C6—C7—O4	−0.8 (2)
C6—C7—C8—C9	1.3 (2)	O4—C7—C8—C9	−179.66 (14)

Symmetry codes: (i) −*x*+1, −*y*, −*z*+1; (ii) −*x*+1, −*y*+1, −*z*+1; (iii) *x*, *y*, *z*+1; (iv) −*x*+1, −*y*+1, −*z*; (v) *x*, *y*, *z*−1.

### Poly[aqua[µ-3-(3,4-dihydroxyphenyl)propenoato]sodium] (2) Hydrogen-bond geometry (Å, º)

**Table:**

*D*—H···*A*	*D*—H	H···*A*	*D*···*A*	*D*—H···*A*
O3—H3···O1^vi^	0.84 (2)	1.84 (2)	2.6437 (19)	158 (2)
O4—H4···O5^vii^	0.81 (2)	1.85 (2)	2.6350 (19)	166 (2)
O5—H5*A*···O1^viii^	0.83 (2)	2.02 (2)	2.826 (2)	163 (2)
O5—H5*B*···O1^i^	0.82 (2)	1.95 (2)	2.7614 (19)	178 (3)

Symmetry codes: (i) −*x*+1, −*y*, −*z*+1; (vi) −*x*+2, −*y*, −*z*; (vii) −*x*, −*y*+1, −*z*; (viii) *x*−1, *y*, *z*.
